# Loss of Cyclin G–Associated Kinase Leads to Lysosome Dysfunction and Immune Modulation in Podocytes

**DOI:** 10.1681/ASN.0000001130

**Published:** 2026-05-18

**Authors:** Patricia Bunda, Xuefei Tian, Soichiro Nagata, Gabriel Lerner, Paulina Medina-Rangel, Jianlei Gu, Hongyu Zhao, Lois E. Greene, Shawn M. Ferguson, Kazunori Inoue, Shuta Ishibe

**Affiliations:** 1 Department of Cellular and Molecular Physiology, Yale School of Medicine, New Haven, Connecticut; 2 Section of Nephrology, Department of Internal Medicine, Yale School of Medicine, New Haven, Connecticut; 3 Department of Pathology, Yale School of Medicine, New Haven, Connecticut; 4 Department of Biostatistics, Yale University, New Haven, Connecticut; 5 Laboratory of Cell Biology, National Heart, Lung, and Blood Institute, National Institutes of Health, Bethesda, Maryland; 6 Department of Cell Biology, Yale School of Medicine, New Haven, Connecticut; 7 Department of Nephrology, The University of Osaka Graduate School of Medicine, Osaka, Japan

**Keywords:** albuminuria, glomerular diseases, podocyte

## Abstract

**Key Points:**

- Loss of podocyte-associated cyclin G–associated kinase resulted in mistrafficking of lysosomal hydrolases.
- Loss of podocyte-associated cyclin G–associated kinase induced immune modulation.

**Background:**

Given the postmitotic nature of podocytes, adapting to both physiologic and pathologic stress is crucial to prevent podocyte loss. An important component of maintaining cellular homeostasis are lysosomes, which are membrane-bound organelles responsible for degradation and recycling of damaged organelles and other macromolecules. Lysosome impairment has been shown to cause cellular and organ dysfunction, highlighting its crucial role in homeostasis.

**Methods:**

We previously showed that podocyte-specific loss of cyclin G–associated kinase knockout (*Gak*-KO) leads to severe proteinuria, podocyte injury, and kidney failure. To interrogate which GAK domains are necessary for its function, we used a transgenic mouse expressing a truncated 62-kDa *C*-terminal GAK protein (GAK C62), which consists of the clathrin-binding and J domains. We evaluated the functional role of GAK C62 in podocytes using immunofluorescence, western blotting, and *in vivo* transcriptomic analysis.

**Results:**

Our findings revealed significant accumulation of autophagic vesicles in *Gak*-KO podocytes. By systematically probing for potential causes of autophagosome accumulation, we showed that loss of *Gak* resulted in impaired lysosomal degradation, secondary to mistrafficking of lysosomal hydrolases. Notably, GAK C62 expression completely rescued these phenotypes at the cellular and organismal levels. Moreover, *in vivo* translating ribosome affinity purification sequencing and cytokine profiling demonstrated enrichment of immune-related pathways and IL-11 production in *Gak*-KO podocytes.

**Conclusions:**

GAK, specifically its *C*-terminal domains, plays an important role in podocyte lysosome homeostasis. Lysosomal dysfunction due to loss of *Gak* led podocytes to adopt immune-like properties characterized by the release of proinflammatory cytokine IL-11.

## Visual Abstract

**Figure:**
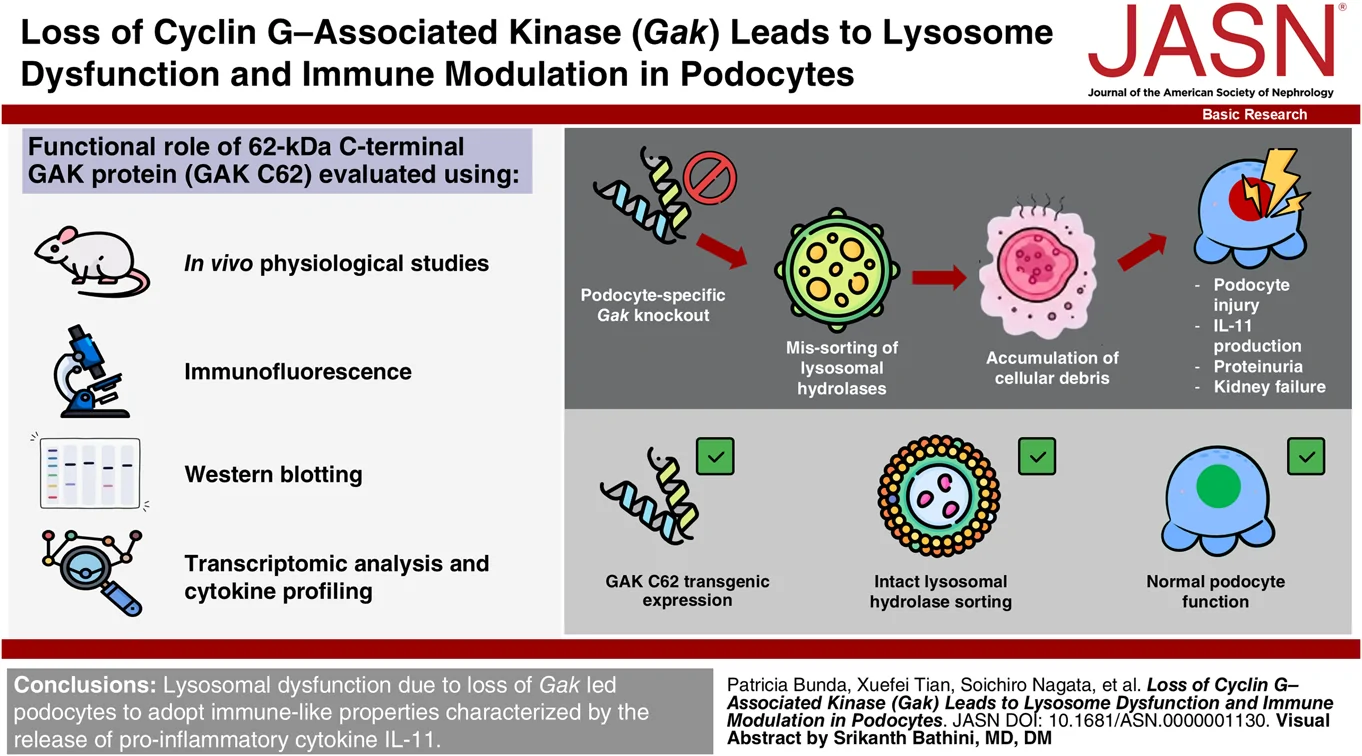

## Introduction

Since Christian de Duve's initial discovery of the lysosome in the 1950s,^1^ this organelle has emerged as a central regulator of cellular homeostasis.^2^ Lysosomes are best known for degrading cellular components, preventing waste accumulation, and generating metabolic intermediates. Their degradative ability relies on the proper function and localization of luminal hydrolases, integral membrane proteins, and membrane-associated proteins. Various pathways, such as endocytosis, phagocytosis, and autophagy, converge to deliver a wide array of cargo to the lysosome, highlighting its central role in intracellular and extracellular turnover.^3^ Furthermore, lysosomes participate in nutrient sensing,^4^ plasma membrane repair,^5^ immunity,^6^ and growth factor signaling.^7^ Consistent with these diverse roles, mutations in genes encoding lysosomal components and regulators lead to cellular and organ dysfunction, as is exemplified in lysosomal storage diseases.^8^

Podocytes are terminally differentiated epithelial cells that line the glomerular basement membrane and are essential for the kidney filtration barrier. These complex cells with interdigitating foot processes are critical for maintaining selectivity. Podocyte loss is a key driver of proteinuria and eventual progression to CKD.^9^ Podocytes experience immense mechanical stress, filtering approximately 180 L of plasma per day,^10^ and their postmitotic nature necessitates adapting to both physiologic and pathologic stress. *In vitro* and *in vivo* animal studies have demonstrated the importance of intact lysosomal function in podocytes.^11–14^ Clinically, patients with lysosomal storage diseases can manifest with significant proteinuria and glomerulosclerosis, accompanied by foot process effacement and morphologic changes in podocytes.^15–17^ Despite these clinical and histologic observations, the mechanisms linking lysosomal dysfunction to podocyte injury and kidney disease remain incompletely understood.

Cyclin G–associated kinase (GAK) is a 160-kDa protein consisting of four domains: an *N*-terminus Ser/Thr kinase domain, a phosphatase and tensin homolog (PTEN)-like domain, a clathrin-binding domain (CB), and a *C*-terminal J domain.^18,19^ GAK is most well known for its role as a cofactor in uncoating of clathrin-coated vesicles from the plasma membrane and trans-Golgi network (TGN),^20^ but it has also been implicated in cell cycle progression,^21,22^ autophagy induction,^23^ and lysosomal function.^24–26^ GAK activity is regulated mainly through protein–protein interactions; its CB and J domains interact with clathrin and heat shock cognate 71kDa protein, mediating clathrin uncoating,^20^ while its interactions with adapter protein complex-1 and adapter protein complex-2 enables cargo sorting at the TGN and plasma membrane, respectively.^25,27^

We previously established the importance of clathrin-mediated endocytosis in podocytes^28,29^ and reported that a subset of patients with CKD exhibited reduced glomerular GAK expression. Podocyte-specific loss of GAK in mice resulted in severe proteinuria and foot process effacement.^30^ Ultrastructural analysis revealed a unique accumulation of autophagic vesicle-like structures in the cell body of cyclin G-associated kinase knockout (*Gak*-KO) podocytes—a phenomenon not observed in other podocyte-specific endocytic knockouts.^28,29^ These findings prompted us to investigate the cell type–specific role of GAK in regulating autophagy and lysosomal function. While earlier studies indicated GAK's involvement in autophagy initiation and lysosome homeostasis, they were predominantly *in vitro,* and the *in vivo* consequences, domain-specific functions, and precise lysosomal defects remain incompletely understood.

In this study, we investigated the role of GAK in regulating autophagy and lysosomal function in podocytes. To define domain-specific functions, we used an *in vivo* model expressing a truncated 62-kDa *C*-terminal fragment of GAK (C62).^31^ Given that the *N*-terminal kinase and PTEN-like domains of GAK are nonessential for its role in clathrin uncoating,^20,32^ we focused specifically on 62-kDa *C*-terminal GAK protein (GAK C62). We hypothesize that the loss of GAK leads to defects in clathrin-mediated endocytosis and TGN-to-lysosome trafficking. Furthermore, we propose that the functional recruitment and uncoating capabilities of GAK C62—consisting of the CB and J domains—are sufficient to maintain these trafficking pathways and preserve the filtration barrier in the absence of the full-length protein.

## Methods

### Generation of Mice

*Gak*^*fl/fl*^ mice on a C57BL/6 background, in which essential coding exon 1 is flanked by *loxP* sites, were obtained from the laboratory of Dr. Lois E. Greene (National Institutes of Health [NIH], Bethesda, MD).^30,33^ The GAK C62 mice on the C57BL/6 background, in which the 62-kDa C-terminal fragment of GAK is expressed from the *Rosa26* promoter, was also a gift from the laboratory of Dr. Lois E. Greene (NIH, Bethesda, MD).^31^ These mice were mated with the *Pod-Cre-Rosa26-Dtr*^*fl*^ mice with a diptheria toxin receptor (DTR) knock-in on a C57BL/6 background, a gift from Lloyd Cantley (Yale University, New Haven, CT),^34^ to generate *Gak*^*fl/fl*^ *Pod-Cre-Rosa-Dtr*^*fl*^ (*Gak*-KO) and GAK C62 *Gak*^*fl/fl*^ *Pod-Cre-Rosa-Dtr*^*fl*^ (C62 *Gak-*KO) mice. *Gak*^*+/+*^ *Pod-Cre-Rosa-Dtr*^*fl*^ were used as wild-type (WT) controls. TRAP mice on a C57BL/6 background were obtained from The Jackson Laboratory (stock 022367). All generated mice were backcrossed for at least five generations before experiments. Mouse tail genotyping was performed by PCR using gene-specific primers (Supplemental Table 3). Sample sizes for *in vivo* experiments (*n*=5 per group) were determined empirically on the basis of prior data from our laboratory and established standards in the field for evaluating podocyte-specific knockout models.^30,35–37^ Given the highly penetrant and robust kidney phenotype of *Gak*-KO mice,^30^ this sample size enabled detection of biologically meaningful differences with relatively small cohort sizes and is statistically sufficient to detect significant differences in structural and functional readouts, including proteinuria and foot process effacement. We also note that group sizes were selected to balance statistical considerations with the ethical principles of animal use reduction, while ensuring reproducibility and interpretability of the observed effects.

### Cell Culture

Primary podocytes were isolated from mice between ages P7–P10 and cultured as previously described in our laboratory.^28,35^ Briefly, whole kidneys were harvested, minced with a scalpel blade, and digested in collagenase A (5 mg/ml) for 20–30 minutes at 37°C. Digested kidney tissues were resuspended in Rosewell Park Memorial Institute (RPMI) 1640 medium and filtered through a 70-*μ*m cell strainer. The filtrates were plated on collagen I–coated dishes and cultured in a 37°C incubator with 5% CO_2_. RPMI 1640 medium is supplemented with 10% FBS, 100 U/ml penicillin/streptomycin, 10 mM HEPES, 1 mM sodium bicarbonate, and 1 mM sodium pyruvate. 0.1 *μ*g/ml diphtheria toxin was added to the RPMI medium, which was changed every other day until only primary podocytes remain (approximately 72 hours). The purity of cultured primary podocytes determined by WT1 immunostaining was >98% (data not shown). Primary podocytes at passage 1 or 2 were used in experiments.

### Translating Ribosome Affinity Purification Sequencing

Podocyte mRNA was isolated from 3- to 4-week-old mice as previously described.^35,38,39^ Resulting mRNA samples were sent to the Yale Center for Genomic Analysis for ribosomal RNA depletion followed by RNA sequencing. Raw fastq files were converted to counts table using a standard pipeline provided by the Yale Center for Genomic Analysis. The resulting counts table was analyzed using differential expression analysis for RNA-Seq data version 2^40^ to obtain differentially expressed genes (DEGs) between samples. Functional analysis was performed using clusterProfiler 4.0^41^ to identify enriched pathways from the list of DEGs.

### Additional Methods

Refer to Supplemental Methods and Supplemental Tables 1–3 for detailed information on antibodies and reagents, kidney histology quantification, ELISA, immunofluorescence, western blotting, transmission electron microscopy (TEM), immuno-electron microscopy, cell adhesion assay, labeled transferrin uptake assay, translating ribosome affinity purification sequencing (TRAP-seq), and cytokine profiling.

### Statistical Analyses

All data are presented as mean±SD unless otherwise noted. The number of replicates for each experiment is shown in the figure legends. Statistical analysis was performed using GraphPad Prism 9.0 software. Statistical comparison between groups was made using one-way or two-way ANOVA, as appropriate, and is shown in the figure legends. *P* values < 0.05 were considered statistically significant.

## Results

### 62-kDa *C*-Terminus of GAK Sufficiently Rescued the Severe Phenotype in *Gak*-KO Mice

As previously reported, podocyte-specific loss of *Gak* in mice resulted in glomerulosclerosis, interstitial fibrosis, and progressive kidney damage.^30^ To determine which domains of *Gak* are essential for its function, we used a transgenic mouse with constitutive, ubiquitous expression of a GAK C62 driven by the *Rosa26* locus.^31^ This truncated GAK protein retains the CB and J domains but lacks the *N*-terminal kinase (K) and PTEN-like (P) domains (Figure 1A). By crossing this mouse with our podocyte-specific *Gak*-KO mouse, we generated the C62 *Gak-*KO mouse and confirmed expression of the truncated protein in primary podocytes by western blot (Figure 1B and Supplemental Figure 1E). Histologic examination revealed marked rescue of the severe *Gak*-KO phenotype in the C62 *Gak-*KO mouse in terms of glomerulosclerosis and interstitial fibrosis (Figure 1, C–E, and Supplemental Figure 1, A–D). GAK C62 expression also restored body weight, urine albumin/creatinine ratio, and plasma creatinine to normal levels (Figure 1, F–H, and Supplemental Figure 1F). Survival of C62 *Gak-*KO mice was comparable with WT controls, whereas *Gak-*KO mice typically survived only approximately 50 days (Figure 1I). Collectively, these results demonstrate that GAK C62 can effectively rescue the severe dysfunction observed in *Gak-*KO mice.

**Figure 1:**
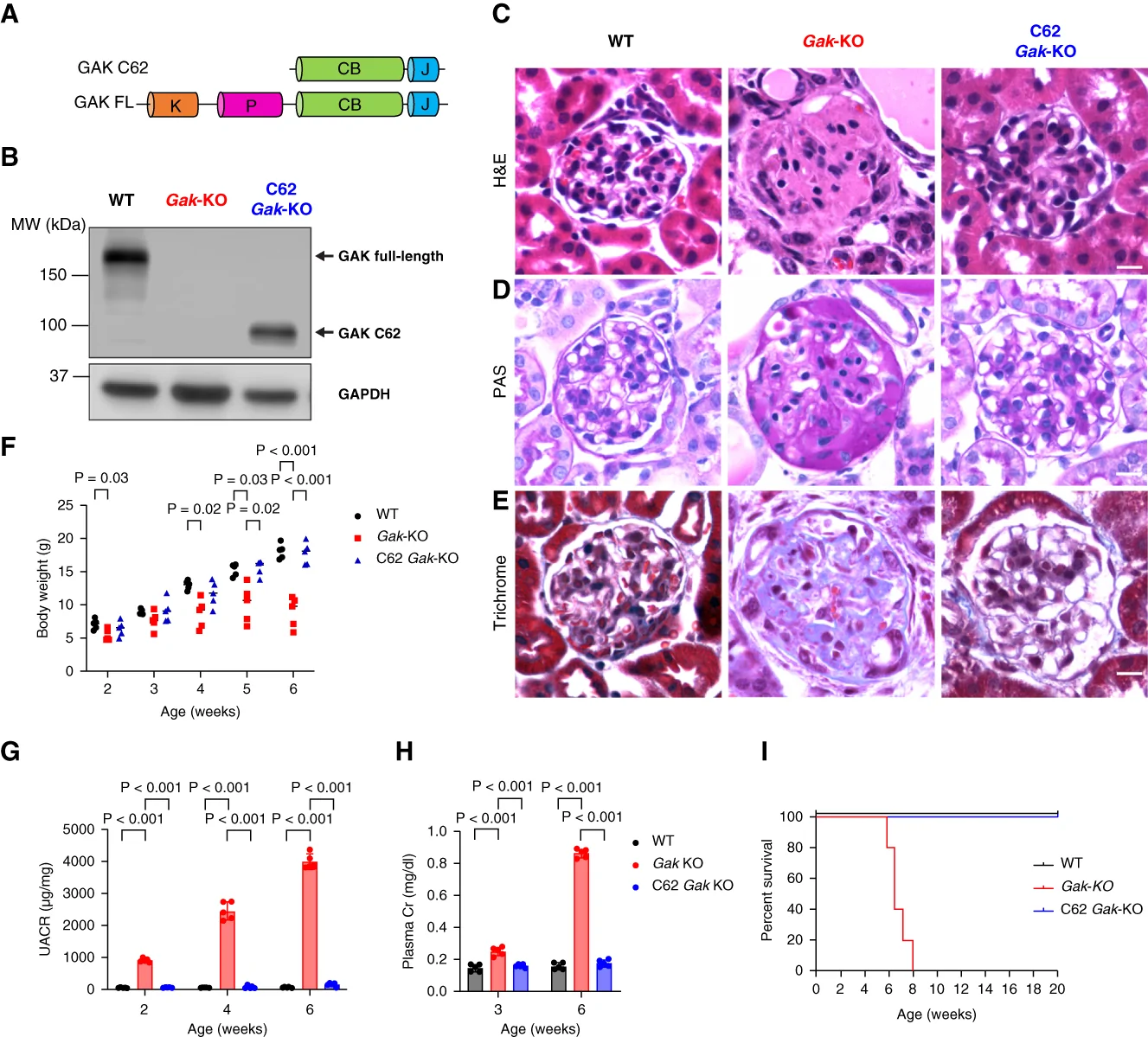
**GAK C62 expression rescues severe proteinuria and kidney failure in *Gak*-KO mice.** (A) Schematic diagram showing GAK C62 and GAK domains. (B) Representative western blot image of GAK expression in cell lysates of primary podocytes isolated from mice age P7–P10, using GAPDH as loading control. (C–E) Representative kidney histologic images obtained by light microscopy using H&E (C), PAS (D), and Masson trichrome (E) staining in WT control, *Gak*-KO, and C62 mouse glomeruli at 6 weeks of age. Scale bar=10 *µ*m. (F) Weekly body weight measurements from 2 to 6 weeks of age. *n*=5 mice. Two-way ANOVA with Tukey multiple comparison test. (G) Quantification of urine albumin normalized to urine creatinine at 2, 4, and 6 weeks of age. *n*=5 mice. Two-way ANOVA with Tukey multiple comparison test. (H) Quantification of plasma creatinine at 3 and 6 weeks of age. *n*=5 mice. Two-way ANOVA with Tukey multiple comparison test. (I) 140-day survival curves of WT control, *Gak*-KO, and C62 mice. *n*=5 mice. Significant *P* values (< 0.05) are shown in the graphs. C62, 62-kDa *C*-terminal fragment of GAK; CB, clathrin-binding domain; Cr, creatinine; FL, full-length; GAK, cyclin G–associated kinase; GAK-C62, 62-kDa *C*-terminal GAK protein; Gak-KO, cyclin G–associated kinase knockout; GAPDH, glyceraldehyde-3-phosphate dehydrogenase; H&E, hemotoxylin and eosin; Hsc70, heat shock cognate 71kDa protein; J, J domain; K, kinase domain; KO, knockout; P, PTEN-like domain; PAS, periodic acid–Schiff; PTEN, phosphatase and tensin homolog; UACR, urine albumin/creatinine ratio; WT, wild-type.

### GAK C62 Is Important in Maintaining Foot Processes and Cell Adhesion in Podocytes

TEM analysis revealed severe foot process effacement in *Gak-*KO mice by 4 weeks of age; however, this was rescued in C62 *Gak-*KO mice (Figure 2, A and B). Because foot process effacement is a compensatory response to podocyte loss, we next quantified podocyte density using a well-established *in vivo* quantification method.^42^ *Gak*-KO mice exhibited a significant reduction in podocyte density per unit glomerular volume (Figure 2, C and D). More importantly, both podocyte density and its inverse measure—glomerular volume per podocyte—demonstrated that GAK C62 expression restored podocyte numbers *in vivo* (Figure 2, D and E). The structural and functional integrity of podocyte foot processes relies on proper cell adhesion onto the extracellular matrix.^43^ Although cell viability was unchanged (data not shown), *Gak-*KO primary podocytes displayed impaired adhesion to collagen type I after trypsinization, which was effectively rescued by GAK C62 expression (Figure 2F).

**Figure 2:**
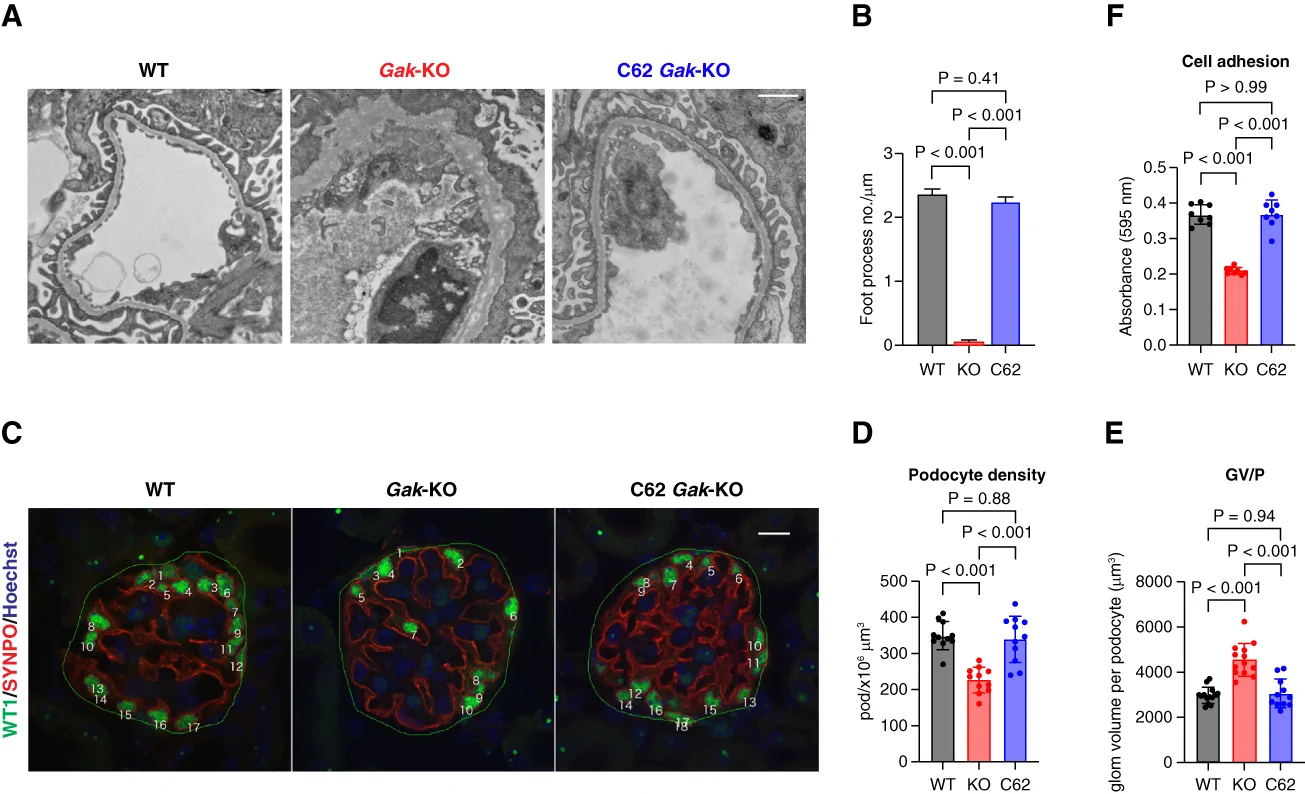
**GAK C62 plays a role in maintaining foot processes and cell adhesion in podocytes.** (A) Representative *in vivo* TEM images of podocyte foot processes at age 4 weeks in WT control, *Gak*-KO, and C62 *Gak*-KO mice. Scale bar=1 *µ*m. (B) Quantification of foot process per unit area in (A). *n*=200. One-way ANOVA. Data are presented as mean±SEM. (C) Representative immunofluorescence images of mouse kidney tissue sections costained with WT1 (green), SYNPO (red), and Hoechst (blue). Outlines demarcate boundaries of the glomerulus (green) and podocyte nuclei (red). Mice are 4 weeks of age. Scale bar=10 *µ*m. (D and E) Quantification of podocyte density per unit volume and its inverse measure—GV/P in (C) using CellProfiler software. *n*=11; each data point represents 8–10 glomeruli. One-way ANOVA with Tukey multiple comparison test. (F) Cell adhesion experiment on *in vitro* primary podocytes quantified by crystal violet staining with absorbance at 595 nm. *n*=8 replicates. One-way ANOVA with Tukey multiple comparison test. GV/P, glomerular volume per podocyte; SYNPO, synaptopodin; TEM, transmission electron microscopy.

### Autophagic Vesicle Accumulation in *Gak*-KO Podocytes Was Alleviated by GAK C62 Expression

In addition to foot process effacement, TEM allowed the assessment of subcellular structures within the podocyte cell body, providing insights into underlying dysfunction.^44^ We observed the presence of round, electron-dense bodies resembling autophagic vesicles in *Gak*-KO podocytes (Figure 3A). Additional TEM imaging revealed abundant single-membrane structures containing partially degraded, nonuniform, electron-dense material (Supplemental Figure 2, A–F), consistent with late autophagic vesicle accumulation.^45^ We also performed immunofluorescence staining on kidney tissues using microtubule-associated protein 1 light chain 3 (LC3), a known marker of autophagic vesicles, and podocyte marker synaptopodin.^46,47^ Our results corroborated the TEM data, demonstrating robust accumulation of LC3 puncta in *Gak-*KO podocytes, which was improved with GAK C62 expression (Figure 3B). These findings were validated *in vitro* using primary podocytes co-immunostained with LC3 and podocyte marker WT1,^48^ where *Gak-*KO podocytes exhibited a marked buildup of LC3-positive puncta, supporting our initial hypothesis of autophagic vesicle accumulation (Figure 3C). Quantification using CellProfiler^49^ revealed increases in both the number and average diameter of LC3-positive puncta per cell in *Gak-*KO podocytes while C62-truncated protein was sufficient to rescue this accumulation (Figure 3, D and E). Immunoblotting further confirmed elevated microtubule-associated proteins 1A/1B light chain 3B, lipidated form (LC3-II) levels in *Gak-*KO podocytes, supporting our *in vivo* and *in vitro* LC3 immunofluorescence findings (Figure 3, F and G). Many LC3-positive puncta colocalized with Ras-related protein Rab-7, a known marker of late endosomes and lysosomes^50^ (Figure 3H and Supplemental Figure 3). Immuno-electron microscopy on *Gak-*KO primary podocytes revealed lysosomal-associated membrane protein 2 (LAMP2)-positive vesicles filled with undegraded cellular material (Supplemental Figure 2, G and H). Finally, systematic analysis and quantification of primary podocyte TEM images corroborate the suggested accumulation of late autophagic vesicles in *Gak*-KO cells (Supplemental Figure 2, I and J).

**Figure 3:**
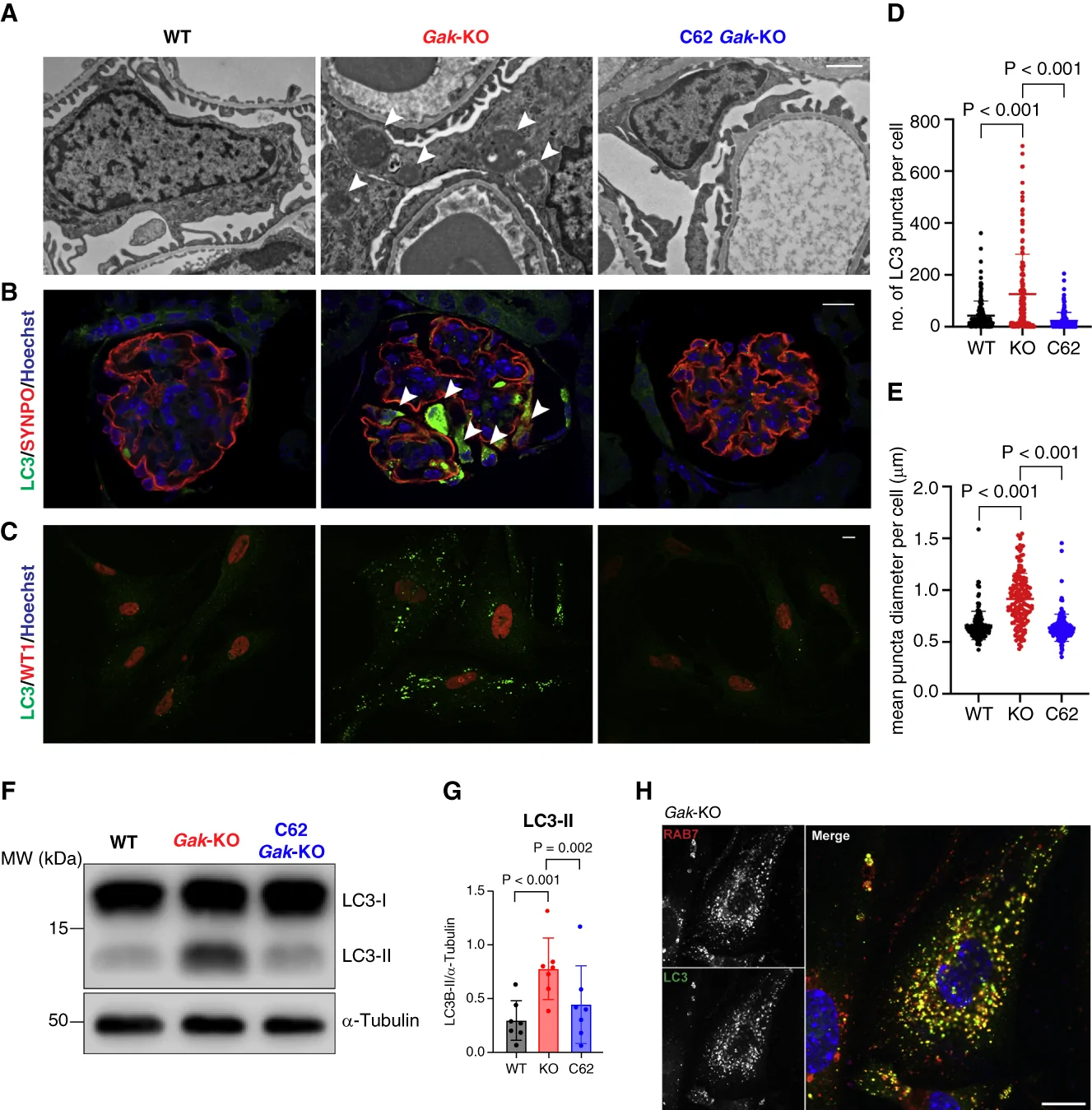
**GAK C62 alleviated autophagic vesicle accumulation due to loss of GAK in podocytes.** (A) TEM images highlighting podocyte cell body at age 4 weeks in WT control, *Gak*-KO, and C62 mice. White arrows highlighting autophagic vesicle-like structures. Scale bar=1 *µ*m. (B) Representative immunofluorescence images of kidney tissue sections costained with SYNPO (red), LC3 (green), and Hoechst (blue). Mice are 4 weeks of age. White arrows highlighting LC3 staining in podocytes. Scale bar=10 *µ*m. (C) Representative immunofluorescence images of *in vitro* primary podocytes costained with WT1 (red), LC3 (green), and Hoechst (blue). Scale bar=10 *µ*m. (D) Quantification of number of LC3 puncta per cell in primary podocytes using CellProfiler software. One-way ANOVA with Tukey multiple comparison test. *n*=160 cells. (E) Quantification of mean LC3 puncta diameter per cell (*µ*m) in primary podocytes using CellProfiler software. One-way ANOVA with Tukey multiple comparison test. *n*=160 cells. (F) Representative western blot showing LC3-I and LC3-II expression in cell lysates of primary podocytes isolated from WT control, *Gak*-KO, and C62 mice (age P7–P10), using *α*-tubulin as loading control. (G) Densitometric analysis of western blot bands in (F) using ImageJ. *n*=8 biologic replicates. One-way ANOVA with Tukey multiple comparison test. (H) Representative immunofluorescence image of *in vitro* primary podocytes costained with RAB7 (red), LC3 (green), and Hoechst (blue). Scale bar=10 *µ*m. Significant *P* values (< 0.05) are shown in the graphs. LC3, light chain 3; LC3-I, microtubule-associated proteins 1A/1B light chain 3B, cytosolic form; LC3-II, microtubule-associated proteins 1A/1B light chain 3B, lipidated form; RAB7, Ras-related protein Rab-7.

### Autophagic Vesicle Accumulation in *Gak-*KO Podocytes Was Not a Result of Upregulated Autophagy

Given that autophagic vesicle accumulation can result from either increased autophagosome production or inadequate degradation by the lysosome,^51^ we next probed whether autophagy induction was upregulated in *Gak*-KO podocytes. In mammalian cells, Unc-51–like kinase-1 (ULK1) functions as the key initiator of autophagy by forming a complex with other proteins to drive induction and is regulated by mammalian target of rapamycin (mTOR) and adenosine monophosphate-activated protein kinase (AMPK) through inhibitory and activating phosphorylation, respectively.^52,53^ We observed no significant difference in phosphorylated-mTOR (Figure 4, A–C). Conversely, phosphorylated AMPK was significantly reduced in *Gak*-KO podocytes (Figure 4, A, D, and E), suggesting suppression of autophagy initiation, which would not account for the observed autophagosome accumulation in *Gak-*KO podocytes. Consistently, AMPK-mediated phosphorylation of ULK1 at Ser555 was significantly decreased in *Gak-*KO podocytes (Figure 4, F–H), whereas mTOR-mediated phosphorylated-ULK1 at Ser757 was unchanged (Figure 4, F, G, and I). Together, these findings indicate that autophagic vesicle accumulation in *Gak*-KO podocytes is unlikely because of enhanced autophagy induction and suggest impaired autophagic degradation.

**Figure 4:**
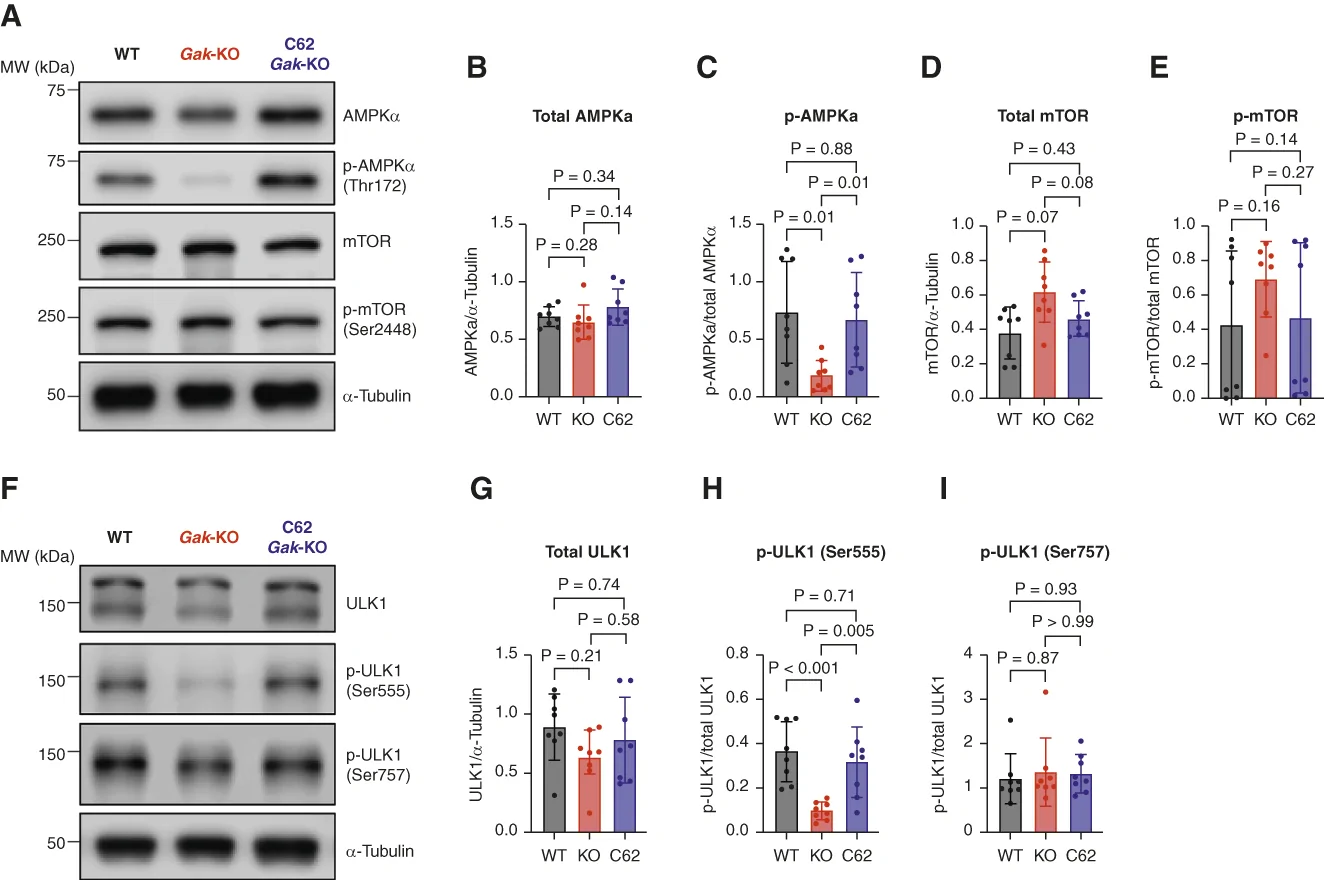
**Loss of *Gak* does not upregulate autophagy induction in podocytes.** (A) Representative western blot images of AMPKa, p-AMPKa (Thr172), mTOR, and phosphorylated-mTOR (Ser2448) expression in cell lysates of primary podocytes isolated from WT control, *Gak*-KO, and C62 mice (age P7–P10), using *α*-tubulin as loading control. (B–E) Densitometric analysis of western blot bands in (A) using ImageJ. *n*=8 biologic replicates. One-way ANOVA with Tukey multiple comparison test. (F) Representative western blot images of ULK1, p-ULK1 (Ser555), and p-ULK1 (Ser757) expression in cell lysates of primary podocytes isolated from WT control, *Gak*-KO, and C62 mice (age P7–P10), using *α*-tubulin as loading control. (G–I) Densitometric analysis of western blot bands in (F) using ImageJ. *n*=8 biologic replicates. One-way ANOVA with Tukey multiple comparison test. AMPKa, adenosine monophosphate-activated protein kinase catalytic subunit alpha; mTOR, mammalian target of rapamycin; p-AMPKa, phophorylated AMPKa; p-ULK1, phosphorylated-Unc-51 like kinase-1.

### Autophagic Flux Measurements Suggest Insufficient Lysosomal Degradation in *Gak*-KO Podocytes

Autophagic flux reflects lysosome-dependent degradation through the autophagy pathway,^54^ which cannot be accurately assessed with static LC3-II measurements levels alone. Therefore, we used the lysosomal inhibitor bafilomycin A1, which inhibits both lysosome acidification and autophagosome–lysosome fusion, effectively preventing LC3-II degradation. Baf A1 treatment conditions were optimized for our podocyte culture system (Supplemental Figure 4). The difference in LC3-II levels in the presence or absence of Baf A1 represents the amount of autophagic material undergoing lysosomal degradation, making it a reliable readout of autophagic flux. Under both Baf A1–treated and untreated conditions, LC3-II levels were higher in *Gak*-KO podocytes compared with WT and C62 *Gak*-KO podocytes (Figure 5, A and B). To further stimulate autophagy, primary podocytes were cultured in Earle's balanced salt solution for 1 hour with or without Baf A1. As expected, starvation increased LC3-II levels relative to nonstarvation conditions (Figure 5, A–C). Absolute autophagic flux, calculated as the difference in LC3-II levels between Baf A1–treated and untreated conditions, was not significantly different across samples (Figure 5, D and E), indicating that lysosomal degradation is occurring in *Gak*-KO podocytes. We also calculated relative flux by normalizing absolute flux to the total starting LC3-II levels, which is represented by the densitometry value under Baf A1 treatment. Despite elevated baseline LC-II levels, the relative flux of *Gak-*KO cells in both RPMI and Earle's balanced salt solution conditions was significantly lower (Figure 5, F and G). These data imply that although autophagic degradation is not fully blocked, the degradative capacity of *Gak*-KO podocytes is insufficient to match the increased autophagic vesicle burden, leading to cytosolic accumulation. Thus, the underlying degradation defect likely results in a functional bottleneck rather than a complete autophagic block.

**Figure 5:**
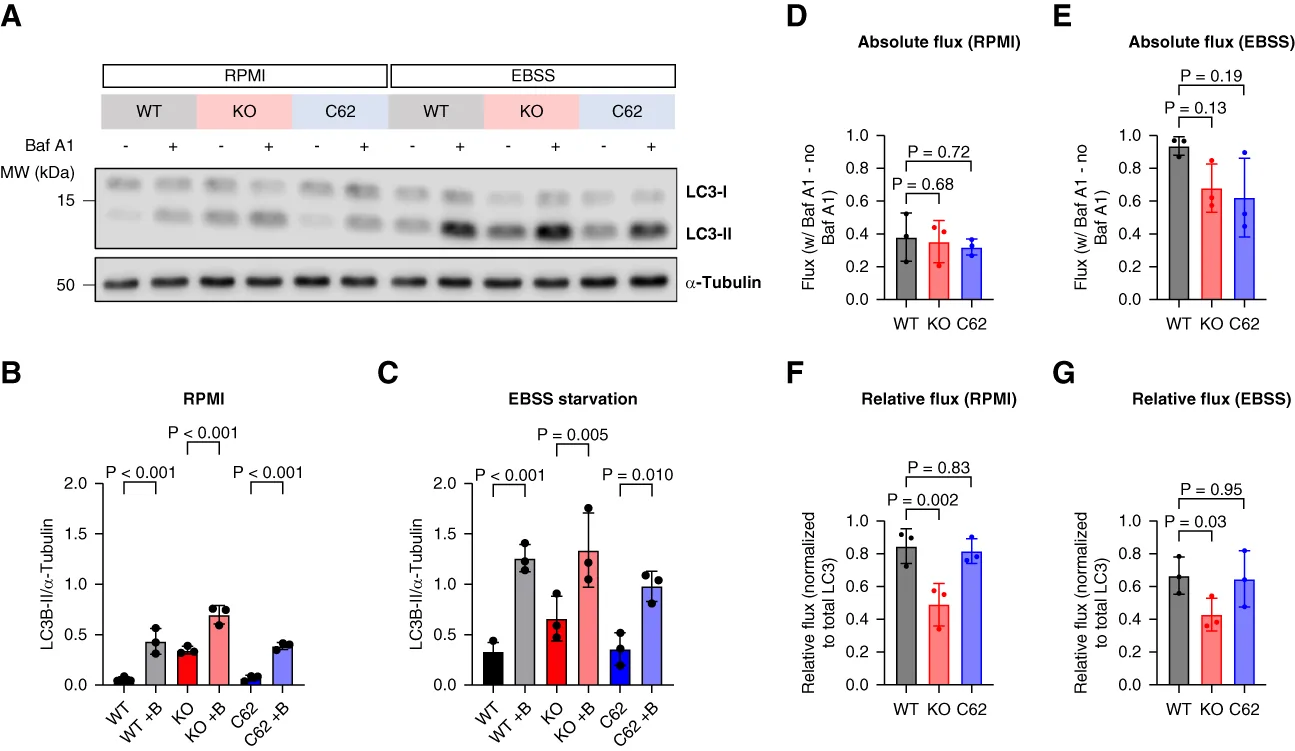
**Loss of *Gak* may result in lysosome-dependent autophagic degradation defect in podocytes.** (A) Representative western blot image showing LC3-I and LC3-II expression in cell lysates of primary podocytes isolated from WT control, *Gak*-KO, and C62 mice (age P7–P10), using *α*-tubulin as loading control. Before lysis, cells were incubated for 1 hour in RPMI (regular media) or EBSS (starvation condition), in the presence or absence of bafilomycin A1 (100 nM). (B and C) Densitometric analysis of western blot bands in (A) using ImageJ. *n*=3 mice. One-way ANOVA with Bonferroni multiple comparison test. (D and E) Absolute autophagic flux measurement using values from (B) and (C) obtained by calculating the difference in LC3-II expression with and without Baf A1 treatment. RM one-way ANOVA with Dunnett multiple comparison test. (F and G) Relative autophagic flux measurement calculated by dividing absolute autophagic flux (D) and (E) by LC3-II expression with Baf A1 treatment (total LC3-II). RM one-way ANOVA with Dunnett multiple comparison test. B, bafilomycin A; EBSS, Earle's balanced salt solution; RM, repeated measures; RPMI, Rosewell Park Memorial Institute.

### Functional Evaluation of the Lysosome Revealed Potential Mistrafficking of Cathepsins

To further define the lysosomal degradation defect, we evaluated multiple aspects of lysosome function. Lysosome acidification was assessed using two complementary fluorescent probes: LysoPrime Green, a pH-insensitive probe that labels all lysosomes, and pHLys Red, a pH-sensitive probe that only labels acidified lysosomes (Dojindo Laboratories). Colocalization of both probes in *Gak*-KO primary podocytes indicated intact lysosomal acidification (Figure 6A). We next examined autophagosome–lysosome fusion by costaining for LC3 and LAMP2, a well-known lysosome marker. Enlarged LC3 puncta in *Gak*-KO podocytes colocalized with LAMP2 (Figure 6B), suggesting intact autophagosome–lysosome fusion, which was further corroborated by the presence of autolysosomes by TEM (Supplemental Figure 2, A–F). In addition, no noticeable difference in the subcellular localization of both LAMP1 and LAMP2 was observed across all genotypes (Supplemental Figure 5). Western blot analysis of LAMP1 and LAMP2 displayed a noticeable shift in apparent molecular weight in *Gak*-KO podocytes (Figure 6, C–E), because of increased *N*-glycosylation (data not shown), which is known to protect LAMP1 and LAMP2 from proteolytic digestion by lysosomal enzymes.^55^ The significant increase in LAMP2 levels in *Gak-*deficient podocytes corroborates the previous findings of late autophagic vesicle accumulation (Supplemental Figure 2, A–H) and highlights its functionally distinct role from LAMP1.^56^ We next analyzed lysosomal cathepsins, which are synthesized as inactive proenzymes and activated by *N*-terminal cleavage during intracellular trafficking.^57^ Immunoblotting revealed reduced levels of mature cathepsins, particularly in *Gak*-KO podocytes (Figure 6, F and G, and Supplemental Figure 6, A and B), while longer exposure showed increased levels of the immature procathepsin B (CTSB) (Figure 6, F and H). This imbalance suggests a defect in intracellular trafficking and maturation of these lysosomal proteases, which may underlie the observed impairment in autophagic degradation in *Gak*-KO podocytes.

**Figure 6:**
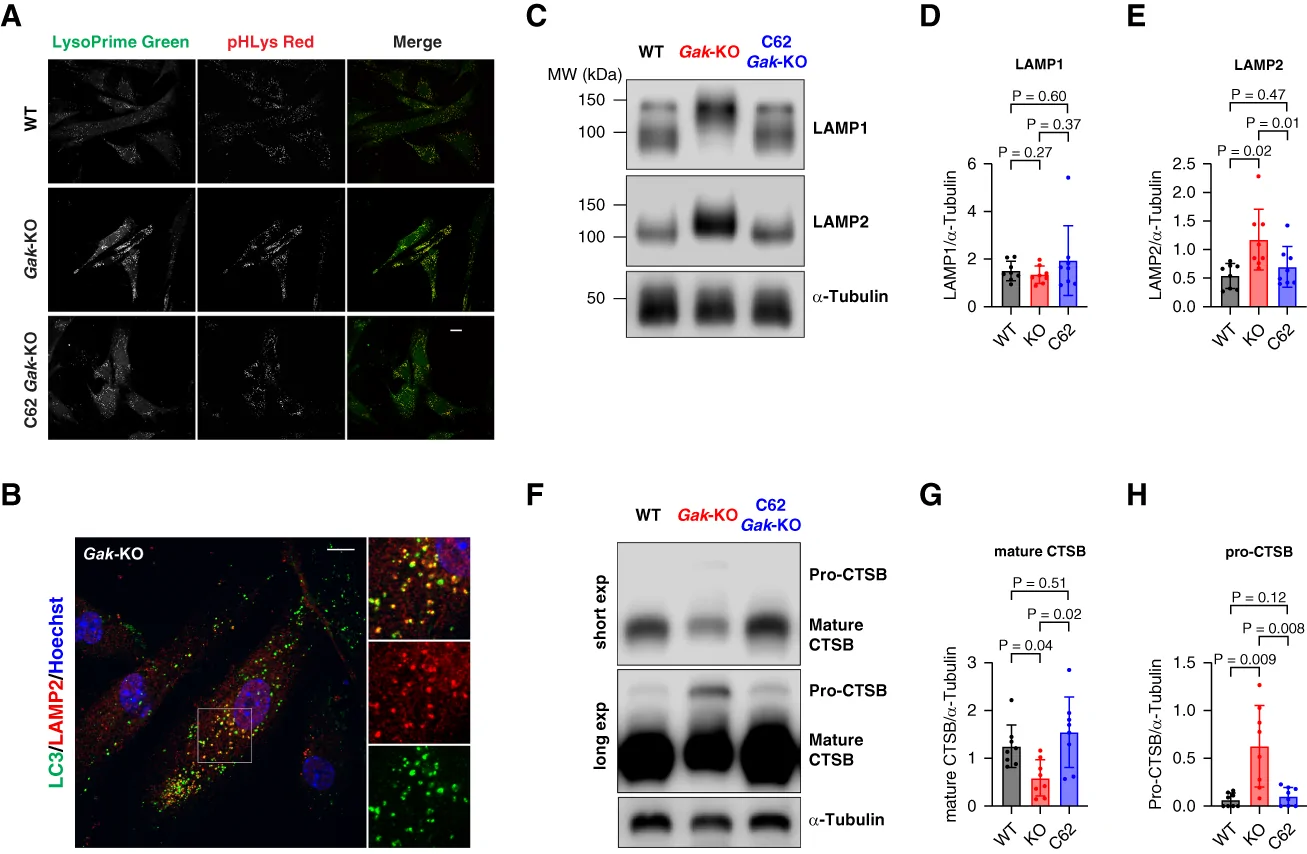
**Lysosome dysfunction in *Gak*-KO podocytes is likely caused by mistrafficking of cathepsins.** (A) Representative immunofluorescence live imaging of *in vitro* primary podocytes using LysoPrime Green and pHLys Red (Dojindo Lysosomal Acidic pH Detection Kit). Scale bar=10 *µ*m. (B) Representative immunofluorescence image of *Gak*-KO *in vitro* primary podocyte costained with LAMP2 (red), LC3 (green), and Hoechst (blue). Scale bar=10 *µ*m. (C) Representative western blot images showing LAMP1 and LAMP2 expression in cell lysates of primary podocytes, using *α*-tubulin as loading control. (D and E) Densitometric analysis of western blot bands in (C) using ImageJ. *n*=8 biologic replicates. One-way ANOVA with Tukey multiple comparison test. (F) Representative western blot images showing CTSB expression in cell lysates of primary podocytes, using *α*-tubulin as loading control. The same CTSB blot was imaged in both short- and long-exposure conditions. (G and H) Densitometric analysis of western blot bands in (F) using ImageJ. *n*=8 biologic replicates. One-way ANOVA with Tukey multiple comparison test. CTSB, cathepsin B; LAMP2, lysosomal-associated membrane protein 2.

### 62-kDa *C*-Terminus of GAK Is Important in Proper Trafficking of Lysosomal Cathepsins

Lysosomal enzymes are delivered from the TGN to lysosomes through clathrin-coated vesicles.^58,59^ Given GAK's role in clathrin uncoating,^20^ we hypothesized that *Gak* deficiency disrupts this process and misroutes lysosomal enzymes. Western blot analysis of concentrated podocyte cell culture supernatants and matched cell lysates revealed that precursor forms of these lysosomal cathepsins were aberrantly secreted into the extracellular space in *Gak* KO cells (Figure 7, A–D). Staining for clathrin light chain demonstrated increased clathrin density in *Gak*-KO podocytes, which was restored by GAK C62 expression (Figure 7, E and F). This is consistent with previous findings that loss of GAK causes clathrin sequestration into immobile cages in the cytosol.^27^ A transferrin time course assay further confirmed impaired clathrin-mediated endocytosis in *Gak*-deficient cells (Supplemental Figure 6D). Moreover, colocalization of clathrin light chain and cathepsin D (CTSD) was significantly reduced in *Gak*-KO podocytes (Figure 7, G and H), indicating defective clathrin-dependent export and delivery of cathepsins from the Golgi. CTSD fluorescence intensity within the Golgi apparatus was used as a surrogate marker of immature cathepsin accumulation (Supplemental Figure 6C). *Gak-*KO cells exhibited higher pro-CTSD intensity relative to WT and C62 *Gak*-KO podocytes (Figure 7I), confirming our prior western blot results. Increased protein levels and fluorescence intensity of cation-independent mannose-6-phosphate receptor in *Gak-*KO podocytes (Supplemental Figure 6, E–G) further supported altered lysosomal hydrolase trafficking. Finally, GAK localization is highly comparable between WT and C62 primary podocytes, characterized by robust perinuclear cytoplasmic staining (Supplemental Figure 6G). This supports the notion that the GAK C62 fragment is appropriately localized to rescue the post-Golgi trafficking of lysosomal cathepsins. Altogether, these findings confirm that GAK C62 expression prevents clathrin sequestration and restores proper lysosomal enzyme trafficking in podocytes.

**Figure 7:**
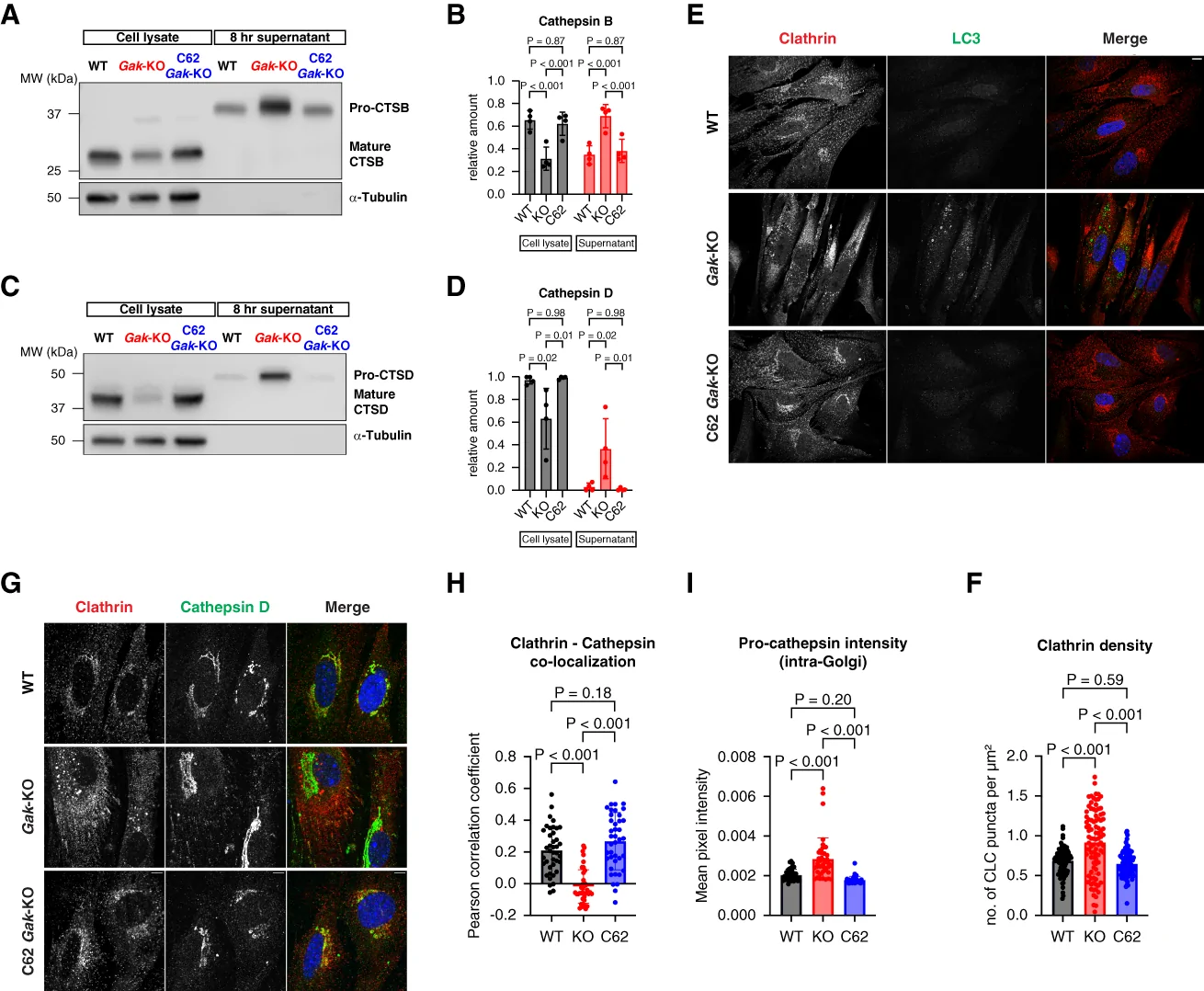
**GAK C62 enables clathrin to participate in proper trafficking of lysosomal cathepsins.** (A) Representative western blot images showing CTSB protein levels in both cell lysates and cell culture supernatant of primary podocytes, using *α*-tubulin as loading control. (B) Densitometric analysis of western blot bands in (A) using ImageJ. Relative amount is calculated by dividing each band by the total amount of cathepsin (cell lysate+cell culture supernatant). *n*=3 biologic replicates. One-way ANOVA with Tukey multiple comparison test. (C) Representative western blot images showing CTSD protein levels in both cell lysates and cell culture supernatant of primary podocytes, using *α*-tubulin as loading control. (D) Densitometric analysis of western blot bands in (C) using ImageJ. Relative amount is calculated by dividing each band by the total amount of cathepsin (cell lysate+cell culture supernatant). *n*=4 biologic replicates. One-way ANOVA with Tukey multiple comparison test. (E) Representative immunofluorescence images of *in vitro* primary podocytes costained with clathrin (red), LC3 (green), and Hoechst (blue). Scale bar=10 *µ*m. (F) Quantification of clathrin density per unit area in (E) using CellProfiler software. *n*=94 cells. One-way ANOVA with Tukey multiple comparison test. (G) Representative immunofluorescence images of *in vitro* primary podocytes costained with clathrin (red), CTSD (green), and Hoechst (blue). Scale bar=10 *µ*m. (H) Quantification of clathrin-cathepsin D colocalization using Pearson correlation coefficient in (G) using CellProfiler software. *n*=40 cells. One-way ANOVA with Tukey multiple comparison test. (I) Quantification of mean intensity of CTSD (intra-Golgi) in (G) using CellProfiler software. *n*=40 cells. One-way ANOVA with Tukey multiple comparison test. CTSD, cathepsin D.

### Loss of *Gak* Upregulated Immune-Related Pathways and IL-11 Production in Podocytes

Having established the role of *Gak* in lysosomal enzyme trafficking, we next examined the broader cellular consequences of lysosome dysfunction using TRAP-seq,^39^ which enables podocyte-specific mRNA profiling *in vivo*. All TRAP-seq samples showed robust transcript levels of podocyte marker genes (Supplemental Figure 7). Unsupervised clustering by principal component analysis and heatmap plots revealed clear transcriptional separation among WT, *Gak*-KO, and C62 *Gak*-KO podocytes at 3–4 weeks of age (Figure 8, A and B). The major axis of variance, PC1, was reflected by differences between *Gak*-KO and WT samples, while the secondary axis of variance, PC2, captured more subtle differences in C62 *Gak*-KO and WT samples (Figure 8A). Differential gene expression analysis using differntial expression analysis for RNA-Seq data version 2^40^ revealed 720 upregulated and 279 downregulated genes in *Gak*-KO and WT samples (p-adj < 0.05) (Supplemental Figures 8, A and B, and 9). By contrast, only eight upregulated and 21 downregulated genes were identified between C62 *Gak*-KO and WT samples (Supplemental Figure 8C), further supporting rescue by GAK C62. Functional enrichment analysis using clusterProfiler 4.0^41^ revealed that the top ten enriched biologic pathways (GO:BP),^60^ between these two groups (Figure 8C), were dominated by interconnected immune-related processes (Figure 8D), aligning with prior reports of podocyte role in innate and adaptive immunity.^61,62^ Because one of the hallmarks of this immune-like phenotype is cytokine secretion,^63,64^ we profiled cytokine levels in primary podocyte supernatants using a Luminex-based multiplex discovery assay (Eve Technologies). Most cytokines and chemokines were undetectable, but this unbiased approach led us to IL-11 (Figure 8, E–J), which was markedly elevated in *Gak-*KO samples (Figure 8J). Given the known role of IL-11 secreted by both podocytes and tubular cells in kidney inflammation and fibrosis,^65^ we further analyzed our TRAP-seq data and found significant upregulation of some IL-11–related transcripts (Supplemental Figure 10, A–F). In addition, IL-11 is known to exert paracrine effects by activating fibroblasts into myofibroblasts, which in turn express *α*-smooth muscle actin (*α*-SMA) and deposit extracellular matrix proteins that contribute to tissue fibrosis.^66^ We observed that *Gak*-KO kidney tissue sections expressed higher levels of IL-11 receptor (IL-11R*α*) and *α*-SMA (Supplemental Figure 10G), supporting a potential role for IL-11 paracrine signaling that may contribute to fibrotic remodeling in our model.

**Figure 8:**
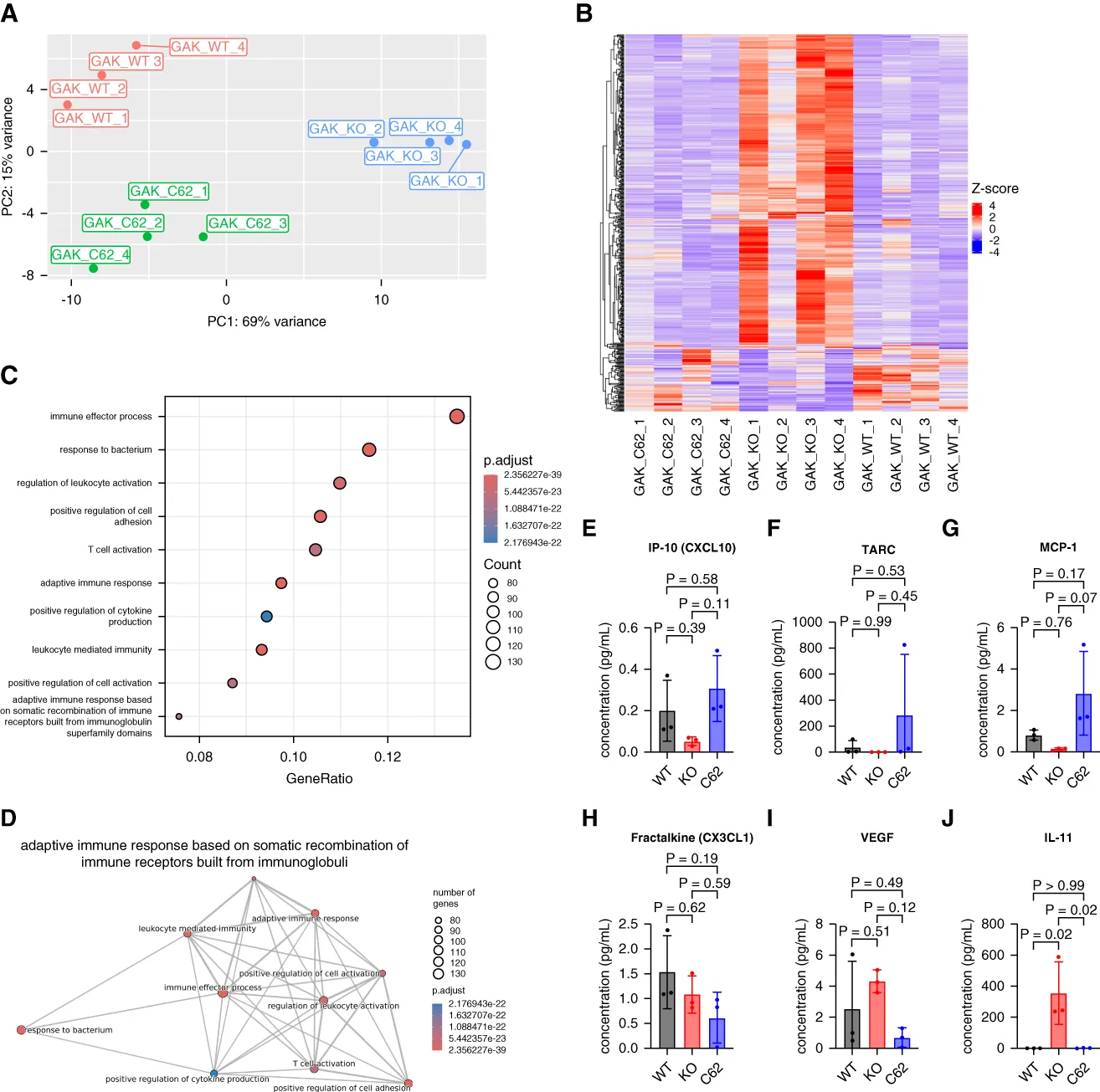
**Loss of *Gak* upregulates immune-related pathways and IL-11 production in primary podocytes in culture.** (A) PCA plot of TRAP-seq samples (four biologic replicates per group) based on normalized transcriptomic counts. (B) Heatmap representing DEGs between *Gak*-KO and WT mouse podocytes across all samples. |log2FC|>1; p-adj < 0.05; 999 total genes. (C) Dot plot and (D) emap plot representing the top ten overrepresented pathways from functional analysis of DEGs between *Gak*-KO and WT mouse podocytes. Analyzed using clusterProfiler and GO:BP database. (E–J) Selected cytokines detected from primary podocyte cell culture supernatant samples using 44-plex Luminex-based multiplex discovery assay (Eve Technologies). *n*=3 mice/biologic replicates. Cytokine concentrations from supernatant were normalized to respective cell lysate concentrations. One-way ANOVA with Tukey multiple comparison test. DEG, differentially expressed gene; GO:BP, Gene Ontology Biological Process; PCA, principal component analysis; TRAP-seq, translating ribosome affinity purification sequencing.

## Discussion

In this study, we identified a novel and critical role for cyclin GAK in regulating lysosome-dependent autophagic degradation in podocytes. Although GAK is classically characterized as a clathrin-uncoating protein essential for endocytosis, ultrastructural and molecular analyses revealed a striking accumulation of autophagic vesicles in *Gak*-KO podocytes—a feature not observed in other knockout models targeting classical endocytic proteins such as dynamin, synaptojanin, endophilin, or epsin. These findings point to a unique and nonredundant function of GAK in the autophagy–lysosome pathway.

Prior *in vitro* studies implicated GAK in maintaining lysosome homeostasis,^24,25^ but the *in vivo* relevance, domain specificity and affected lysosomal functions remained unclear. In this study, we demonstrate, using complementary *in vivo* and *in vitro* models, that GAK C62 is indispensable for maintaining podocyte lysosome-mediated autophagic degradation. Remarkably, the GAK C62 fragment could fully rescue autophagic vesicle accumulation in *Gak-*KO podocytes. Systematic mechanistic analysis revealed that this phenotype arises from defective lysosomal enzyme trafficking. Our findings align with prior observations in nonpodocyte systems where clustered regularly interspaced short palindromic repeats/Cas9–mediated knockout of GAK in A549 cells exhibited accumulation of enlarged autophagic vesicles.^26^ However, important differences emerged as they reported impaired maturation of cathepsin B, with preserved levels of mature CTSD, whereas our data revealed mislocalization of both cathepsins in *Gak*-KO podocytes. Furthermore, whereas earlier studies suggested impaired autophagosome–lysosome fusion and lysosomal acidification, we observed both processes autophagosome-lysosome fusion and preserved lysosomal acidification to be intact, indicating cell type–specific differences in GAK function.

Two siRNA-based studies in HeLa cells further proposed a kinase-dependent role of GAK in lysosomal enzyme sorting.^24,25^ Given that the GAK C62 fragment, which lacks the kinase domain, sufficiently rescues the cathepsin trafficking defect in *Gak*-KO podocytes, this suggests that the kinase and PTEN-like domains of GAK may be dispensable in certain cell types. Likewise, prior studies in HeLa cells reporting altered ULK1 signaling after GAK depletion was not recapitulated in podocytes, suggesting differences in experimental models and depletion strategies and cell type–specific compensation mechanism.^23^ Consistent with this, *in vivo* studies expressing kinase-dead form of GAK, resulted in neonatal lethality due to pulmonary dysfunction, secondary to disorganized lung architecture.^67^ By contrast, expression of the GAK C62 fragment in the liver was sufficient to rescue the lethality and liver dysfunction due to loss of GAK in hepatocytes.^31,33^ Furthermore, given GAK's implications as a risk factor of Parkinson's disease,^68^ this warrants further investigation.

The impact of impaired lysosomal degradation may extend beyond autophagy because TRAP-seq revealed that *Gak*-KO podocytes adopt an immune-like transcriptional program. On the basis of previous studies,^69^ we hypothesize that inadequate degradation of cellular debris due to lysosome dysfunction contributes to podocyte stress and injury, triggering the release of damage-associated molecular patterns (DAMPs). Notably, *Gak*-KO podocytes express higher levels of pattern recognition receptors (Supplemental Figure 11), which recognize DAMPs and initiate inflammatory signaling.^70,71^ IL-11, a profibrotic and proinflammatory cytokine,^66^ was found to be highly upregulated in *Gak*-KO primary podocytes in culture, which may reflect a response to heightened intracellular DAMPs. Whether IL-11 secretion by podocytes is a direct result of GAK loss or generic injury response requires further investigation. Additional experiments such as direct modulation of IL-11 signaling, temporal profiling, and comparative analyses with other injury models are needed to delineate the specific contribution of GAK loss to immune-like activation in podocytes. Notably, IL-11 has been implicated in podocyte and tubular cell–driven fibrosis in mouse model of Alport syndrome,^65^ where anti–IL-11 treatment contributed to prolonged survival and reduction in kidney fibrosis and inflammation. Currently, there are three active phase 2 clinical trials investigating the effect of IL-11 signaling inhibition as a therapeutic target for lung fibrosis,^72^ emphasizing its potential translational relevance.

While our findings provide deep mechanistic insights, some limitations remain. We did not directly assess GAK-dependent phosphorylation or fully resolve the relationship between its kinase-dependent and clathrin-mediated functions. In addition, kinetic analysis of lysosomal trafficking and the Golgi structure may help define the precise site and mechanism of trafficking disruption. The relevance of these findings to human nephrotic syndromes remains uncertain, and further validation in human glomerular disease tissue is needed. Although our data implicate defective lysosomal trafficking as a central mechanism, the molecular intermediates linking clathrin dysregulation to DAMP production remain undefined. Moreover, while GAK loss profoundly reduces the availability of free clathrin monomers required for vesicle trafficking from both the plasma membrane and the TGN, it remains unclear whether lysosomal defects driven by cathepsin mistrafficking are the primary driver of podocyte dysfunction or whether concomitant impaired clathrin-mediated endocytosis plays a more dominant pathogenic role.

In conclusion, our study establishes GAK as an essential regulator of lysosomal enzyme trafficking and autophagic homeostasis in podocytes. The unique phenotype observed in *Gak*-KO mice underscores GAK's nonredundant function in maintaining podocyte integrity. These findings expand the biological scope of GAK beyond clathrin uncoating and reveal the mechanistic link between lysosomal dysfunction, podocyte injury, and immune activation, with important implications for targeting proteinuric kidney diseases.

## Supplementary Material

## Data Availability

All data needed to evaluate the conclusions of the study are present in the main text and the Supplemental Material. Original data generated for the study are available in a public access repository. Data Type: Raw Data/Source Data. Repository Name: Gene Expression Omnibus. Both raw and processed RNA-Seq data are deposited in the Gene Expression Omnibus database (GSE302106).

## Declarative Statements

All animal experiments were conducted in accordance with the NIH Guide for the Care and Use of Laboratory Animals or an equivalent standard that meets or exceeds the ethical and welfare requirements outlined in the NIH Guide. All protocols were approved by the appropriate institutional animal care and use committee.

## Supplemental Material

Supplemental Figure 1. Loss of *Gak* in podocytes results in severe glomerulosclerosis and interstitial fibrosis.

Supplemental Figure 2. *Gak* loss results in accumulation of autophagic vesicles filled with undegraded cellular material.

Supplemental Figure 3. Loss of *Gak* in podocytes results in increased RAB7 puncta size and colocalization with LC3.

Supplemental Figure 4. Optimization of autophagic flux assay conditions in primary podocytes.

Supplemental Figure 5. Loss of *Gak* does not affect lysosome positioning in podocytes.

Supplemental Figure 6. Reduction in cathepsins hydrolases in *Gak*-KO podocytes is due to impaired trafficking from the TGN.

Supplemental Figure 7. Podocyte marker genes have robust transcript levels in TRAP-seq samples.

Supplemental Figure 8. TRAP-seq reveals DEGs in *Gak*-KO and C62 *Gak*-KO podocytes relative to WT controls.

Supplemental Figure 9. Plots highlighting DEGs between *Gak*-KO and WT control podocytes.

Supplemental Figure 10. Investigating potential autocrine and paracrine effects of IL-11 in primary podocytes.

Supplemental Figure 11. Expression levels of pattern recognition receptor genes from TRAP-seq.

Supplemental Table 1. List of primary antibodies and appropriate dilutions used across all experiments.

Supplemental Table 2. List of secondary antibodies and appropriate dilutions used across all experiments.

Supplemental Table 3. List of genotyping primer sequences.
