# Supplementary material for: Loss of Cyclin G–Associated Kinase Leads to Lysosome Dysfunction and Immune Modulation in Podocytes
Source: J Am Soc Nephrol. 2026 May 18;37(11):2435–49. doi: 10.1681/ASN.0000001130 (PMC13742993; doi:10.1681/ASN.0000001130)
Supplement: Supplementary file 1 [file jasn-37-2435-s001.pdf]

ASN American Society of Nephrology | JASN Journal of the American Society of Nephrology | KIDNEY360 Accessing Our World From Every Angle | CJASN Clinical Journal of the American Society of Nephrology

ASN Journal Disclosure Form

As per ASN journal policy, I have disclosed any financial relationships or commitments I have held in the past 36 months as included below. I have listed my Current Employer below to indicate there is a relationship requiring disclosure. If no relationship exists, my Current Employer is not listed.

P. Bunda has nothing to disclose.

I understand that the information above will be published within the journal article, if accepted, and that failure to comply and/or to accurately and completely report the potential financial conflicts of interest could lead to the following: 1) Prior to publication, article rejection, or 2) Post-publication, sanctions ranging from, but not limited to, issuing a correction, reporting the inaccurate information to the authors' institution, banning authors from submitting work to ASN journals for varying lengths of time, and/or retraction of the published work.

Name: Patricia Bunda
Manuscript ID: JASN-2025-000924R1
Manuscript Title: Loss of cyclin G-associated kinase (Gak) leads to lysosome dysfunction and immune modulation in podocytes
Date of Completion: December 12, 2025
Disclosure Updated Date: December 12, 2025

ASN American Society of Nephrology JASN Journal of the American Society of Nephrology KIDNEY360 Accessing Our World From Every Angle CJASN Clinical Journal of the American Society of Nephrology

ASN Journal Disclosure Form

As per ASN journal policy, I have disclosed any financial relationships or commitments I have held in the past 36 months as included below. I have listed my Current Employer below to indicate there is a relationship requiring disclosure. If no relationship exists, my Current Employer is not listed.

S. Ferguson has nothing to disclose.

I understand that the information above will be published within the journal article, if accepted, and that failure to comply and/or to accurately and completely report the potential financial conflicts of interest could lead to the following: 1) Prior to publication, article rejection, or 2) Post-publication, sanctions ranging from, but not limited to, issuing a correction, reporting the inaccurate information to the authors' institution, banning authors from submitting work to ASN journals for varying lengths of time, and/or retraction of the published work.

Name: Shawn M Ferguson
Manuscript ID: JASN-2025-000924R1
Manuscript Title: Loss of cyclin G-associated kinase (Gak) leads to lysosome dysfunction and immune modulation in podocytes
Date of Completion: December 18, 2025
Disclosure Updated Date: December 18, 2025

ASN American Society of Nephrology | JASN Journal of the American Society of Nephrology | KIDNEY360 Accessing Our World From Every Angle | CJASN Clinical Journal of the American Society of Nephrology

ASN Journal Disclosure Form

As per ASN journal policy, I have disclosed any financial relationships or commitments I have held in the past 36 months as included below. I have listed my Current Employer below to indicate there is a relationship requiring disclosure. If no relationship exists, my Current Employer is not listed.

L. Greene reports the following:
Employer: National Institutes of Health

I understand that the information above will be published within the journal article, if accepted, and that failure to comply and/or to accurately and completely report the potential financial conflicts of interest could lead to the following: 1) Prior to publication, article rejection, or 2) Post-publication, sanctions ranging from, but not limited to, issuing a correction, reporting the inaccurate information to the authors' institution, banning authors from submitting work to ASN journals for varying lengths of time, and/or retraction of the published work.

Name: Lois Greene
Manuscript ID: JASN-2025-000924R1
Manuscript Title: Loss of cyclin G-associated kinase (Gak) leads to lysosome dysfunction and immune modulation in podocytes
Date of Completion: December 15, 2025
Disclosure Updated Date: December 15, 2025

ASN American Society of Nephrology JASN Journal of the American Society of Nephrology KIDNEY360 Accessing Our World From Every Angle CJASN Clinical Journal of the American Society of Nephrology

ASN Journal Disclosure Form

As per ASN journal policy, I have disclosed any financial relationships or commitments I have held in the past 36 months as included below. I have listed my Current Employer below to indicate there is a relationship requiring disclosure. If no relationship exists, my Current Employer is not listed.

J. Gu reports the following:
Employer: Yale University

I understand that the information above will be published within the journal article, if accepted, and that failure to comply and/or to accurately and completely report the potential financial conflicts of interest could lead to the following: 1) Prior to publication, article rejection, or 2) Post-publication, sanctions ranging from, but not limited to, issuing a correction, reporting the inaccurate information to the authors' institution, banning authors from submitting work to ASN journals for varying lengths of time, and/or retraction of the published work.

Name: Jianlei Gu
Manuscript ID: JASN-2025-000924R1
Manuscript Title: Loss of cyclin G-associated kinase (Gak) leads to lysosome dysfunction and immune modulation in podocytes
Date of Completion: December 12, 2025
Disclosure Updated Date: November 2, 2025

ASN Journal Disclosure Form

As per ASN journal policy, I have disclosed any financial relationships or commitments I have held in the past 36 months as included below. I have listed my Current Employer below to indicate there is a relationship requiring disclosure. If no relationship exists, my Current Employer is not listed.

K. Inoue reports the following:
Employer: The University of Osaka Graduate School of Medicine; and Speakers Bureau: Mochida pharmaceutical Co. Ltd. and Kowa pharmaceutical Co. Ltd.

I understand that the information above will be published within the journal article, if accepted, and that failure to comply and/or to accurately and completely report the potential financial conflicts of interest could lead to the following: 1) Prior to publication, article rejection, or 2) Post-publication, sanctions ranging from, but not limited to, issuing a correction, reporting the inaccurate information to the authors' institution, banning authors from submitting work to ASN journals for varying lengths of time, and/or retraction of the published work.

Name: Kazunori Inoue
Manuscript ID: JASN-2025-000924R3
Manuscript Title: Loss of Cyclin G–Associated Kinase (Gak) Leads to Lysosome Dysfunction and Immune Modulation in Podocytes
Date of Completion: April 22, 2026
Disclosure Updated Date: April 19, 2026

ASN Journal Disclosure Form

As per ASN journal policy, I have disclosed any financial relationships or commitments I have held in the past 36 months as included below. I have listed my Current Employer below to indicate there is a relationship requiring disclosure. If no relationship exists, my Current Employer is not listed.

S. Ishibe reports the following:
Employer: Yale University School of Medicine; Consultancy: Bristol Myers Squibb; Honoraria: Bristol Myers Squibb; and Other Interests or Relationships: Kidney 360 Associate Editor.

I understand that the information above will be published within the journal article, if accepted, and that failure to comply and/or to accurately and completely report the potential financial conflicts of interest could lead to the following: 1) Prior to publication, article rejection, or 2) Post-publication, sanctions ranging from, but not limited to, issuing a correction, reporting the inaccurate information to the authors' institution, banning authors from submitting work to ASN journals for varying lengths of time, and/or retraction of the published work.

Name: Shuta Ishibe
Manuscript ID: JASN-2025-000924R1
Manuscript Title: "Loss of cyclin G-associated kinase (Gak) leads to lysosome dysfunction and immune modulation in podocytes
Date of Completion: December 18, 2025
Disclosure Updated Date: December 12, 2025

ASN Journal Disclosure Form

As per ASN journal policy, I have disclosed any financial relationships or commitments I have held in the past 36 months as included below. I have listed my Current Employer below to indicate there is a relationship requiring disclosure. If no relationship exists, my Current Employer is not listed.

G. Lerner reports the following:
Employer: Eastern Connecticut Pathology Consultants

I understand that the information above will be published within the journal article, if accepted, and that failure to comply and/or to accurately and completely report the potential financial conflicts of interest could lead to the following: 1) Prior to publication, article rejection, or 2) Post-publication, sanctions ranging from, but not limited to, issuing a correction, reporting the inaccurate information to the authors' institution, banning authors from submitting work to ASN journals for varying lengths of time, and/or retraction of the published work.

Name: Gabriel B. Lerner
Manuscript ID: JASN-2025-000924R2
Manuscript Title: Loss of cyclin G-associated kinase (Gak) leads to lysosome dysfunction and immune modulation in podocytes
Date of Completion: April 9, 2026
Disclosure Updated Date: April 9, 2026

ASN American Society of Nephrology
JASN Journal of the American Society of Nephrology
KIDNEY360 Accessing Our World From Every Angle
CJASN Clinical Journal of the American Society of Nephrology

ASN Journal Disclosure Form

As per ASN journal policy, I have disclosed any financial relationships or commitments I have held in the past 36 months as included below. I have listed my Current Employer below to indicate there is a relationship requiring disclosure. If no relationship exists, my Current Employer is not listed.

P. Medina Rangel reports the following:
Employer: Yale University

I understand that the information above will be published within the journal article, if accepted, and that failure to comply and/or to accurately and completely report the potential financial conflicts of interest could lead to the following: 1) Prior to publication, article rejection, or 2) Post-publication, sanctions ranging from, but not limited to, issuing a correction, reporting the inaccurate information to the authors' institution, banning authors from submitting work to ASN journals for varying lengths of time, and/or retraction of the published work.

Name: Paulina Medina Rangel
Manuscript ID: JASN-2025-000924R2
Manuscript Title: Loss of cyclin G-associated kinase (Gak) leads to lysosome dysfunction and immune modulation in podocytes
Date of Completion: April 9, 2026
Disclosure Updated Date: April 9, 2026

ASN American Society of Nephrology | JASN Journal of the American Society of Nephrology | KIDNEY360 Accessing Our World From Every Angle | CJASN Clinical Journal of the American Society of Nephrology

ASN Journal Disclosure Form

As per ASN journal policy, I have disclosed any financial relationships or commitments I have held in the past 36 months as included below. I have listed my Current Employer below to indicate there is a relationship requiring disclosure. If no relationship exists, my Current Employer is not listed.

S. Nagata has nothing to disclose.

I understand that the information above will be published within the journal article, if accepted, and that failure to comply and/or to accurately and completely report the potential financial conflicts of interest could lead to the following: 1) Prior to publication, article rejection, or 2) Post-publication, sanctions ranging from, but not limited to, issuing a correction, reporting the inaccurate information to the authors' institution, banning authors from submitting work to ASN journals for varying lengths of time, and/or retraction of the published work.

Name: Soichiro Nagata
Manuscript ID: JASN-2025-000924R1
Manuscript Title: Loss of cyclin G-associated kinase (Gak) leads to lysosome dysfunction and immune modulation in podocytes
Date of Completion: December 12, 2025
Disclosure Updated Date: September 23, 2025

ASN American Society of Nephrology | JASN Journal of the American Society of Nephrology | KIDNEY360 Accessing Our World From Every Angle | CJASN Clinical Journal of the American Society of Nephrology

ASN Journal Disclosure Form

As per ASN journal policy, I have disclosed any financial relationships or commitments I have held in the past 36 months as included below. I have listed my Current Employer below to indicate there is a relationship requiring disclosure. If no relationship exists, my Current Employer is not listed.

X. Tian reports the following:
Employer: Yale University

I understand that the information above will be published within the journal article, if accepted, and that failure to comply and/or to accurately and completely report the potential financial conflicts of interest could lead to the following: 1) Prior to publication, article rejection, or 2) Post-publication, sanctions ranging from, but not limited to, issuing a correction, reporting the inaccurate information to the authors' institution, banning authors from submitting work to ASN journals for varying lengths of time, and/or retraction of the published work.

Name: Xuefei Tian
Manuscript ID: JASN-2025-000924R1
Manuscript Title: Loss of cyclin G-associated kinase (Gak) leads to lysosome dysfunction and immune modulation in podocytes
Date of Completion: December 12, 2025
Disclosure Updated Date: March 11, 2025

ASN American Society of Nephrology
JASN Journal of the American Society of Nephrology
KIDNEY360 Accessing Our World From Every Angle
CJASN Clinical Journal of the American Society of Nephrology

ASN Journal Disclosure Form

As per ASN journal policy, I have disclosed any financial relationships or commitments I have held in the past 36 months as included below. I have listed my Current Employer below to indicate there is a relationship requiring disclosure. If no relationship exists, my Current Employer is not listed.

H. Zhao reports the following:
Employer: Yale University

I understand that the information above will be published within the journal article, if accepted, and that failure to comply and/or to accurately and completely report the potential financial conflicts of interest could lead to the following: 1) Prior to publication, article rejection, or 2) Post-publication, sanctions ranging from, but not limited to, issuing a correction, reporting the inaccurate information to the authors' institution, banning authors from submitting work to ASN journals for varying lengths of time, and/or retraction of the published work.

Name: Hongyu Zhao
Manuscript ID: JASN-2025-000924R1
Manuscript Title: Loss of cyclin G-associated kinase (Gak) leads to lysosome dysfunction and immune modulation in podocytes
Date of Completion: January 2, 2026
Disclosure Updated Date: November 5, 2025
