# Supplementary material for: Loss of Cyclin G–Associated Kinase Leads to Lysosome Dysfunction and Immune Modulation in Podocytes
Source: J Am Soc Nephrol. 2026 May 18;37(11):2435–49. doi: 10.1681/ASN.0000001130 (PMC13742993; doi:10.1681/ASN.0000001130)
Supplement: Supplementary file 2 [file jasn-37-2435-s002.pdf]

Supplemental Material for

Loss of Cyclin G–Associated Kinase (*Gak*) Leads to Lysosome Dysfunction and Immune Modulation in Podocytes

Patricia Bunda *et al.*

Corresponding author email: shuta.ishibe@yale.edu

Table of Contents

Supplemental Figure 1. Loss of *Gak* in podocytes results in severe glomerulosclerosis and interstitial fibrosis

Supplemental Figure 2. *Gak* loss results in accumulation of autophagic vesicles filled with undegraded cellular material

Supplemental Figure 3. Loss of *Gak* in podocytes results in increased RAB7 puncta size and co-localization with LC3

Supplemental Figure 4. Optimization of autophagic flux assay conditions in primary podocytes

Supplemental Figure 5. Loss of *Gak* does not affect lysosome positioning in podocytes

Supplemental Figure 6. Reduction in cathepsins hydrolases in *Gak*-KO podocytes is due to impaired trafficking from the TGN

Supplemental Figure 7. Podocyte marker genes have robust transcript levels in TRAP-seq samples

Supplemental Figure 8. TRAP-seq reveals differentially expressed genes (DEGs) in *Gak*-KO and C62 *Gak*-KO podocytes relative to WT control

Supplemental Figure 9. Plots highlighting DEGs between *Gak*-KO and WT control podocytes

Supplemental Figure 10. Investigating potential autocrine and paracrine effects of IL-11 in primary podocytes

Supplemental Figure 11. Expression levels of pattern recognition receptor genes from TRAP-seq

Supplemental Table 1. List of primary antibodies and appropriate dilutions used across all experiments

Supplemental Table 2. List of secondary antibodies and appropriate dilutions used across all experiments

Supplemental Table 3. List of genotyping primer sequences

Supplemental Methods

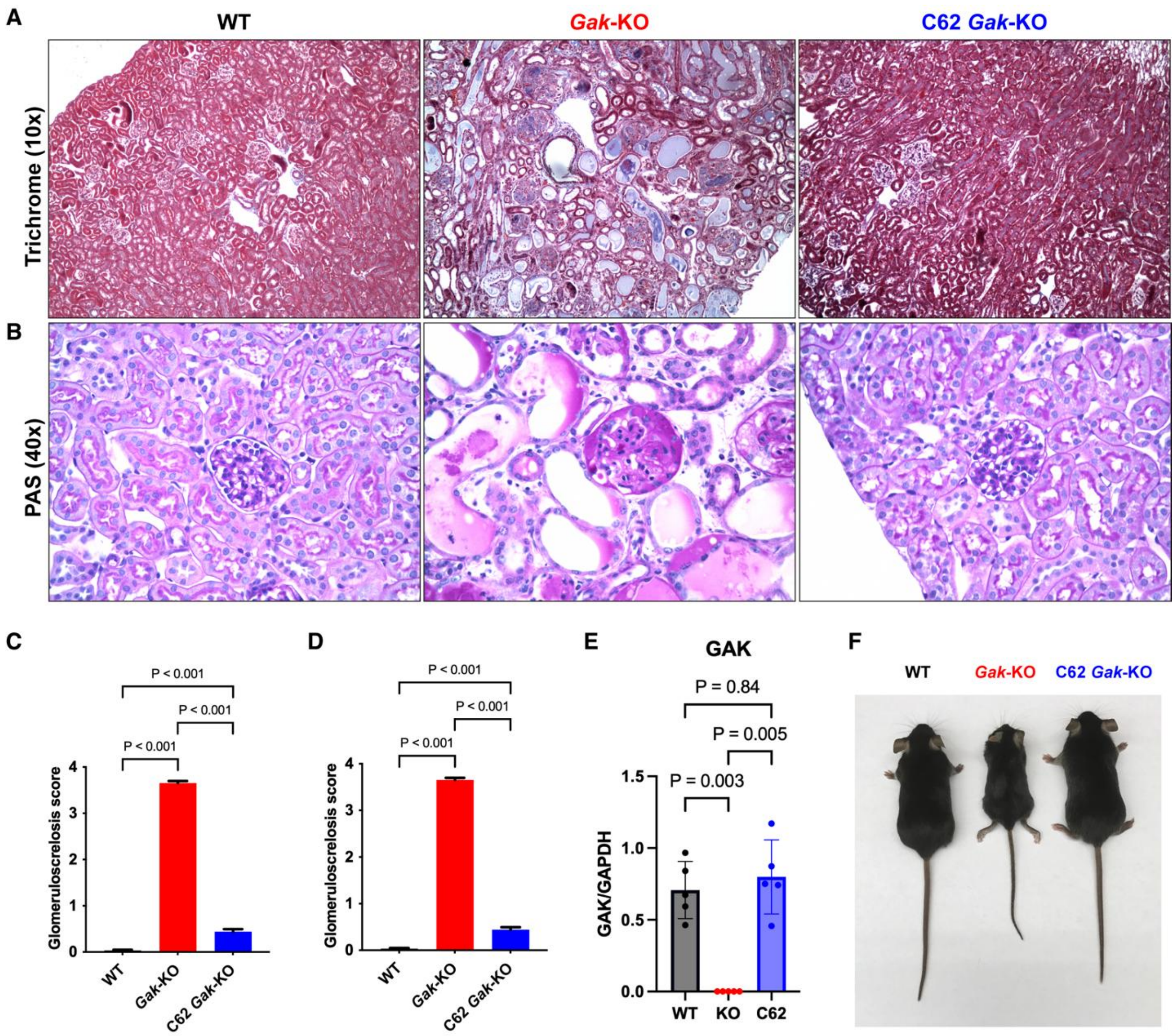

Supplemental Figure 1. Loss of *Gak* in podocytes results in severe glomerulosclerosis and interstitial fibrosis

(A) Representative light microscopy histology images using Masson's Trichrome staining in wildtype control, *Gak*-KO, and C62 mouse kidney interstitial area at 6 weeks of age. 10x magnification. **(B)** Representative light microscopy histology images using Periodic acid-Schiff (PAS) staining in wildtype control, *Gak*-KO, and C62 mouse glomerular area at 6 weeks of age. 40x magnification. **(C)** Quantification of interstitial fibrosis score in (A). n = 5 mice. One-way ANOVA with Tukey's multiple comparison test. Data are presented as mean ± SEM. **(D)** Quantification of glomerulosclerosis score in (B). n = 5 mice. One-way ANOVA with Tukey's multiple comparison test. Data are presented as mean ± SEM. **(E)** Densitometric analysis of western blot bands in Figure 1B using ImageJ. n =5 biological replicates (mice). One-way ANOVA with Tukey's multiple comparison test. **(F)** Representative image of wildtype control, *Gak*-KO, and C62 mice at 6 weeks of age.

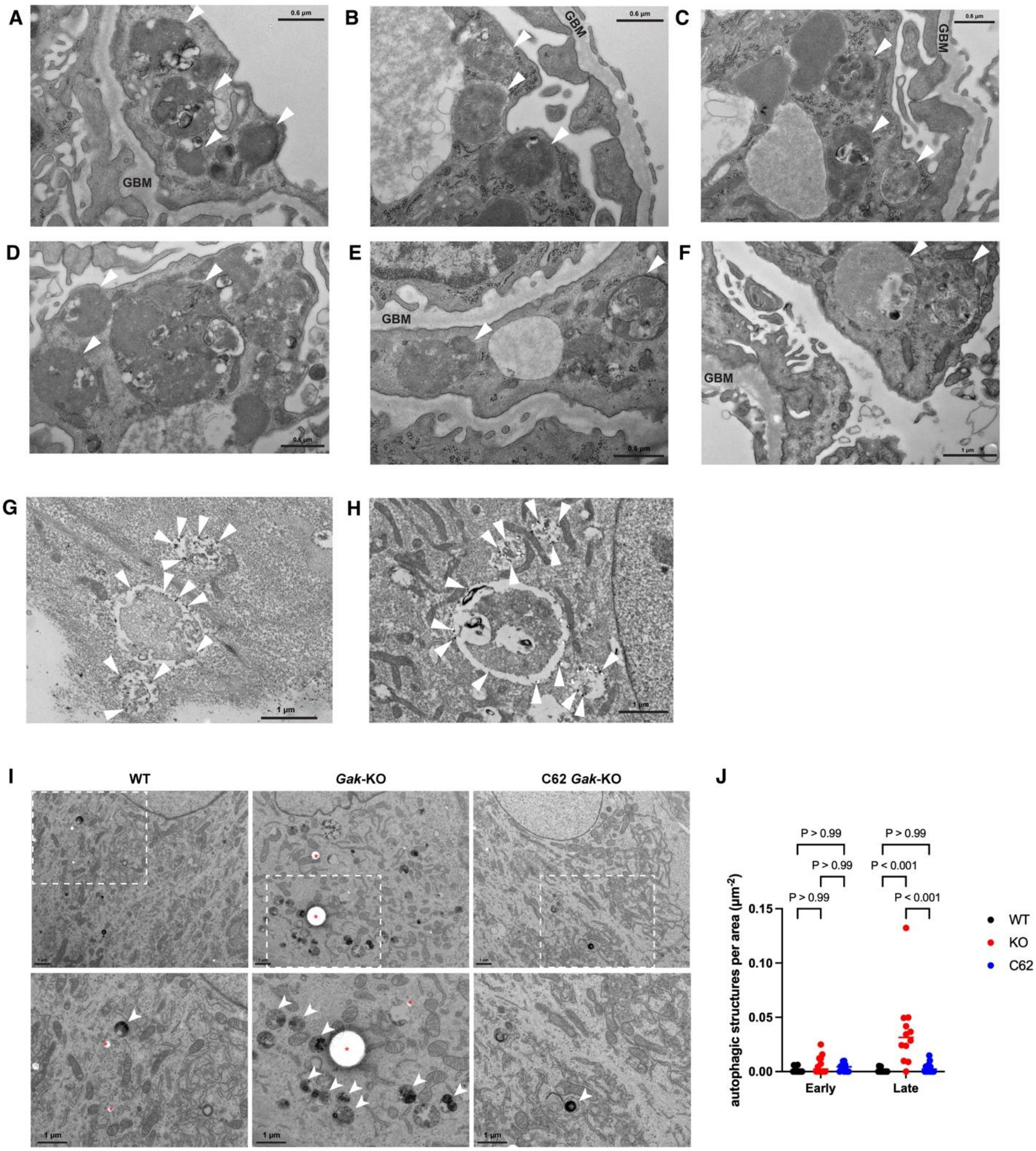

Supplemental Figure 2. *Gak* loss results in accumulation of autophagic vesicles filled with undegraded cellular material

(A-F) Additional TEM images of several *Gak*-KO podocytes *in vivo*. White arrows highlighting autophagic vesicles. GBM: glomerular basement membrane. **(G-H)** Representative immuno-EM images using anti-LAMP2 antibody on *Gak*-KO primary podocytes *in vitro*. White arrows highlighting immunogold particle labeling. **(I)** Representative TEM images of WT, *Gak*-KO, and C62 *Gak*-KO podocytes perinuclear area *in vitro*. White arrowheads: late autophagic vesicles; red asterisks: imaging artifact from small holes on TEM film. **(J)** Quantification of early versus

late autophagic structures in (I) based on published morphological criteria ($n = 12$, total area ~3,000 μm^2 per genotype across multiple podocytes). Two-way ANOVA with Bonferroni's multiple comparison test.

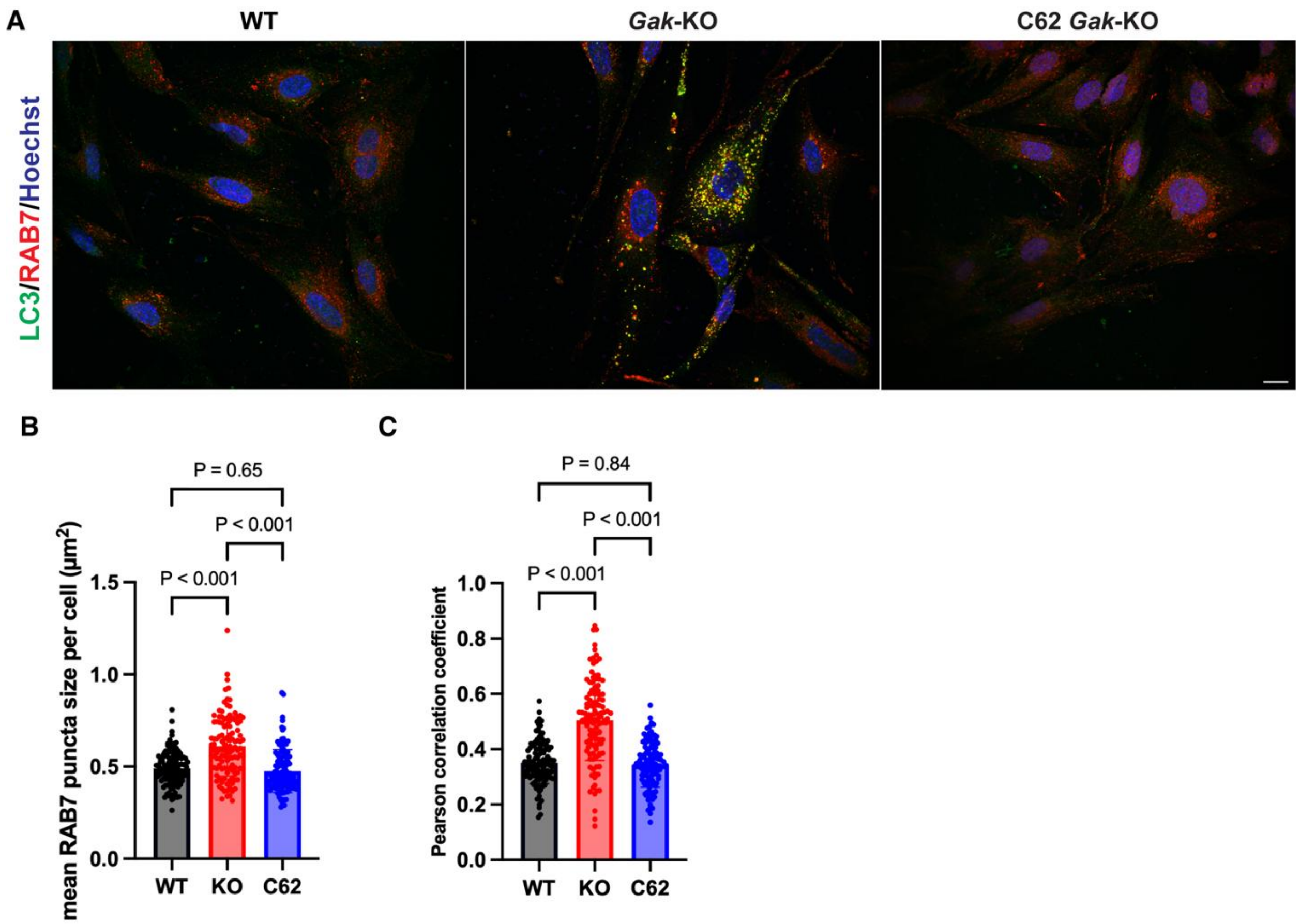

Supplemental Figure 3. Loss of *Gak* in podocytes results in increased RAB7 puncta size and co-localization with LC3

(A) Representative immunofluorescence image of *in vitro* primary podocytes co-stained with RAB7 (red), LC3 (green), and Hoechst (blue). Scale bar = 10 μm. **(B)** Quantification of mean RAB7 puncta diameter per cell (μm) in (A) using CellProfiler software. n = 118 cells. One-way ANOVA with Tukey's multiple comparison test. **(C)** Quantification of RAB7-LC3 co-localization using Pearson Correlation Coefficient in (A) using CellProfiler software. n = 118 cells. One-way ANOVA with Tukey's multiple comparison test.

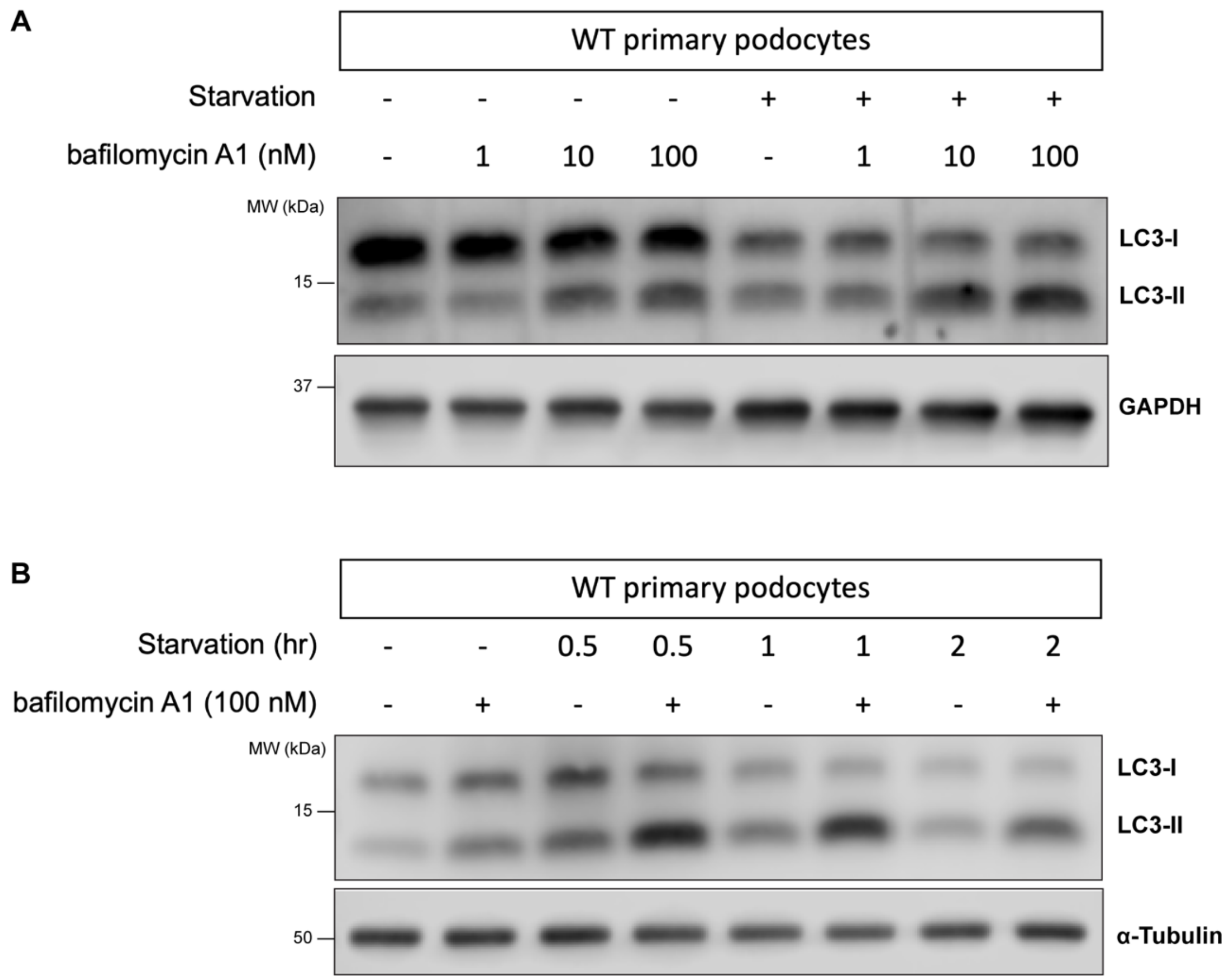

Supplemental Figure 4. Optimization of autophagic flux assay conditions in primary podocytes

(A) Western blot image showing LC3-I and LC3-II expression in cell lysates of primary podocytes isolated from wildtype control mice. Prior to lysis, cells were incubated for 1 hour in RPMI (regular media) or amino acid- and serum-free media (starvation condition), in the presence or absence of bafilomycin A1 at various concentrations. GAPDH was used as loading control. **(B)** Western blot image showing LC3-I and LC3-II expression in cell lysates of primary podocytes isolated from wildtype control mice. Prior to lysis, cells were incubated in RPMI (regular media) or EBSS (starvation condition) at 0, 0.5, 1, and 2-hour durations, in the presence or absence of 100 nM bafilomycin A1. α-Tubulin was used as loading control.

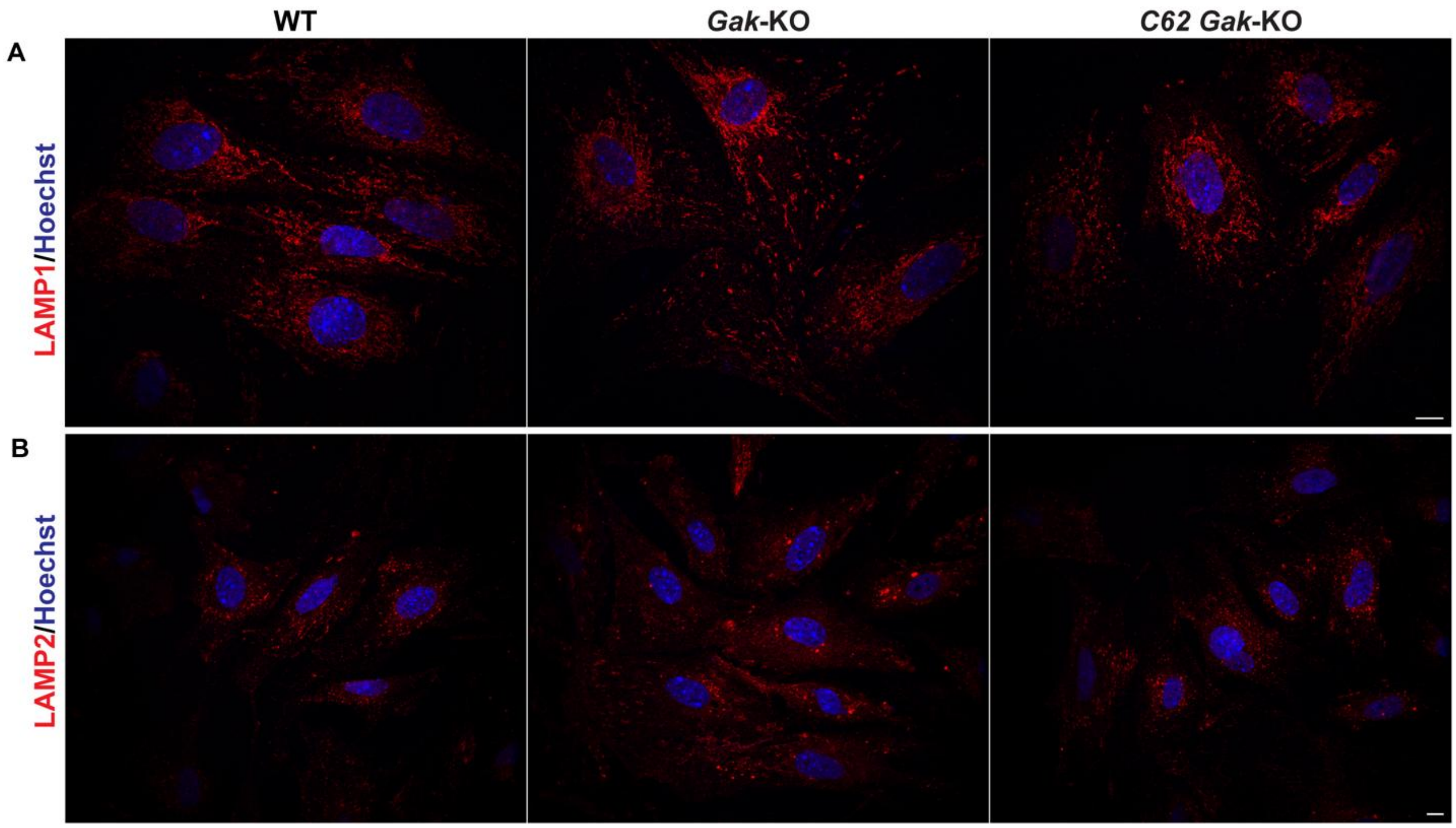

Supplemental Figure 5. Loss of *Gak* does not affect lysosome positioning in podocytes
(A) Representative immunofluorescence images of *in vitro* primary podocytes co-stained with LAMP1 (red) and Hoechst (blue). Scale bar = 10 µm. **(B)** Representative immunofluorescence images of *in vitro* primary podocytes co-stained with LAMP2 (red) and Hoechst (blue). Scale bar = 10 µm.

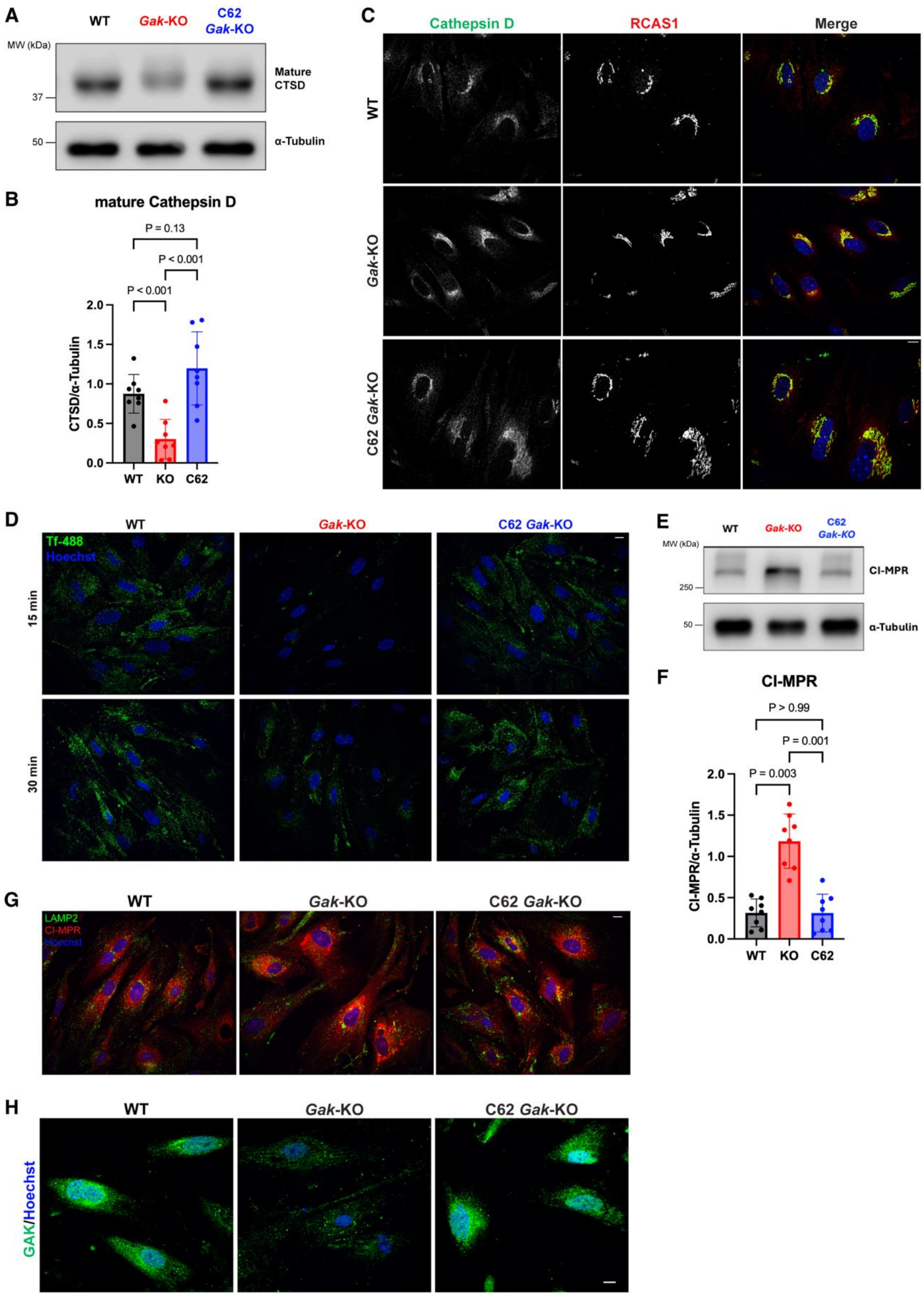

Supplemental Figure 6. Reduction in cathepsins hydrolases in *Gak*-KO podocytes is due to impaired trafficking from the TGN

(A) Representative western blot images showing cathepsin D (CTSD) expression in cell lysates of primary podocytes, using α-Tubulin as loading control. **(B)** Densitometric analysis of western blot bands in (A) using ImageJ. n= 8 biological replicates. One-way ANOVA. **(C)** Representative immunofluorescence images of *in vitro* primary podocytes co-stained with RCAS1 (red), cathepsin D (green), and Hoechst (blue). Scale bar = 10 µm. **(D)** Representative images from labeled Transferrin-488 (green) assay at 15- and 30-minute timepoints. Hoechst (blue) is used as a nuclear marker. Scale bar = 10 µm. **(E)** Representative western blot images showing cation-independent mannose-6-phosphate receptor (CI-MPR) expression in cell lysates of primary podocytes, using α-Tubulin as loading control. **(F)** Densitometric analysis of western blot bands in (D) using ImageJ. n= 8 biological replicates. One-way ANOVA. **(G)** Representative immunofluorescence image of *in vitro* primary podocytes co-stained with CI-MPR (red), LAMP2 (green), and Hoechst (blue). Scale bar = 10 µm. **(H)** Representative immunofluorescence image of *in vitro* primary podocytes co-stained with GAK (green) and Hoechst (blue). Scale bar = 10 µm.

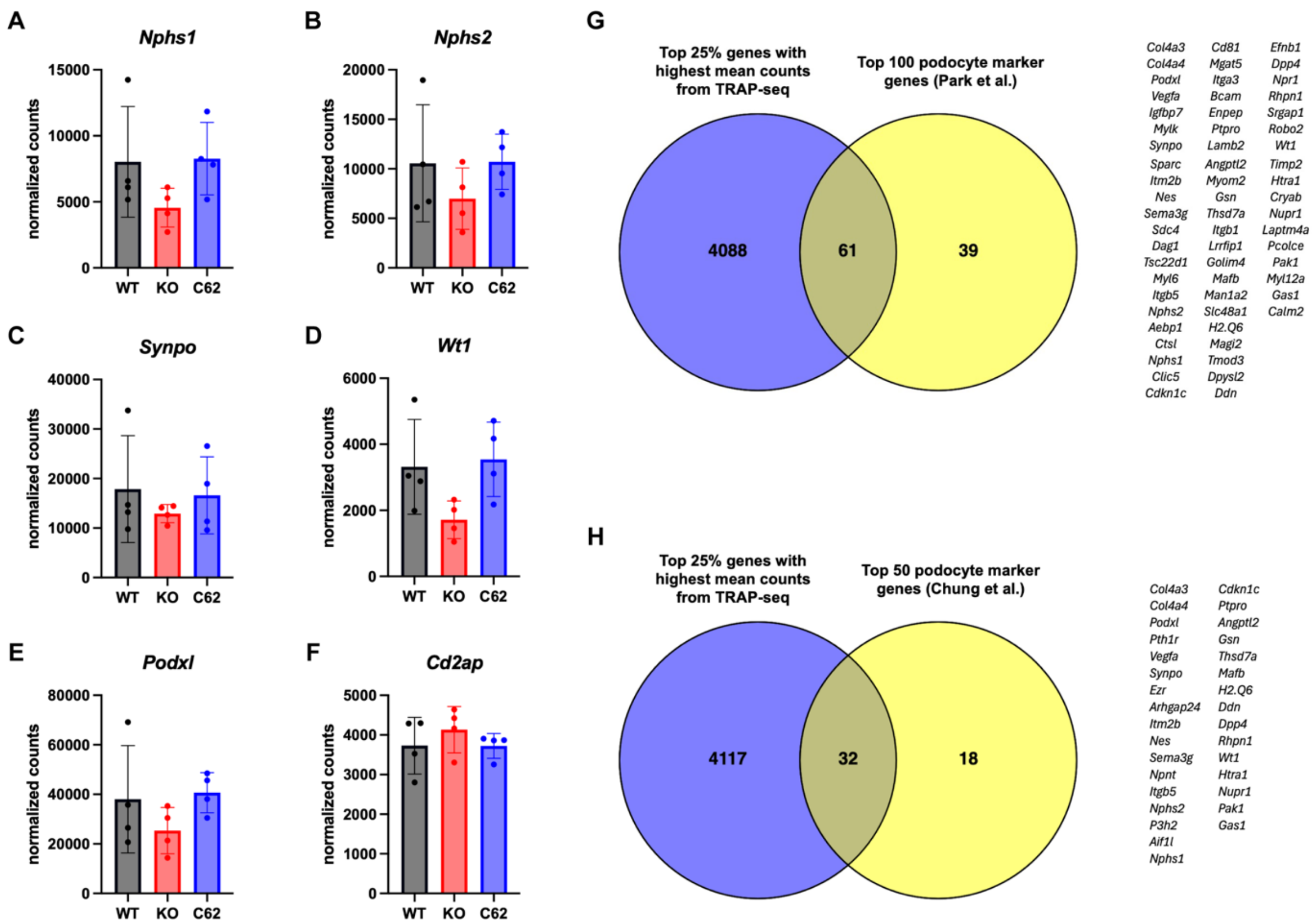

Supplemental Figure 7. Podocyte marker genes have robust transcript levels in TRAP-seq samples

(A-F) Gene expression normalized counts of podocyte markers from TRAP-seq experiment. Normalized using DESeq2. n = 4 biological replicates. **(G-H)** Venn diagram showing the overlap between top 25% highest mean counts from TRAP-seq and curated podocyte marker genes from two scRNA-seq datasets. Overlapped genes are listed on the right of corresponding Venn diagram.

A

Gak-KO vs WT, Top 30 upregulated DEGs

SYMBOL	log2FoldChange	padj
Klk1b21	19.90836138	0.003695935
Crlf1	6.675338756	4.00E-11
Lcn2	5.660864911	1.77E-42
Col17a1	5.039069738	0.023863234
C3	4.86354718	1.12E-66
Serpina3n	4.756972315	3.28E-06
Serpina10	4.548161014	4.36E-20
Lgi2	4.245877951	2.04E-09
Nfasc	4.091452544	0.04036396
Pgbd5	4.00583371	0.008575878
Timp1	4.002104951	8.82E-07
Ubd	3.962456684	1.90E-05
Cxcl2	3.959302463	0.000507161
Il1rn	3.957820173	1.92E-07
Ccdc33	3.937491421	0.005579043
Epha6	3.919779159	0.000347182
Lif	3.891225822	3.91E-12
Fcrl2	3.838795406	0.008780167
Cacna1s	3.838042303	0.000185664
Rplp0-ps1	3.797336169	0.01816483
Fgg	3.782388721	0.006018123
Ltbp2	3.62819501	3.02E-17
Rgs16	3.593229988	4.57E-05
Itgam	3.526820062	2.82E-13
Sorcs1	3.396983115	0.007032064
Krt20	3.38896393	6.77E-10
Ubb-ps	3.321748326	0.000364152
Serpine1	3.298083071	9.35E-17
Acsbg1	3.286602113	3.85E-07
Havcr1	3.174516612	0.000717428

B

Gak-KO vs WT, Top 30 downregulated DEGs

SYMBOL	log2FoldChange	padj
Strc	-29.21698519	0.000000875
Slco1a1	-10.26035112	1.46E-12
Kdm5d	-10.0593118	2.06E-03
Eif2s3y	-9.308749479	0.004187236
Uty	-8.944314715	4.42E-03
Ddx3y	-6.710626968	1.03E-02
Pzp	-4.119864218	9.24E-06
Acsm3	-3.425705876	2.57E-02
Slc7a13	-3.325316296	5.51E-14
0610031O16Rik	-3.200532267	0.0000108
Slc22a7	-2.602099492	1.88E-03
Slc22a30	-2.574118724	1.74E-14
Itih2	-2.41837032	0.004294107
Cyp4b1	-2.302513014	3.71E-14
Mgat5b	-2.250958665	0.022059193
Slc13a5	-2.227202147	0.016157361
Gm10167	-2.212737979	4.78E-02
Tbx10	-2.175255498	0.002965263
Gm2602	-2.170956088	0.0000856
Ces1f	-2.160638451	0.000000352
Mfsd2a	-2.138411711	0.0000605
Gja3	-2.054301987	3.07E-03
Gm15348	-1.966589745	1.50E-03
BC049987	-1.955674151	1.52E-03
Akr1c14	-1.904838835	0.021917624
Gbp10	-1.746109496	6.49E-04
Gm9361	-1.724713233	0.000417554
Dcst1	-1.71825657	7.48E-03
Gm21057	-1.67312661	3.45E-02
Slc22a28	-1.649014402	0.000000035

C

C62 *Gak*-KO vs WT, Total DEGs

SYMBOL	log2FoldChange	padj
Rps13-ps1	3.801031624	0.025617186
Gm9917	1.568446774	1.47E-05
Depp1	1.313838808	1.58E-02
Ttr	1.207931472	0.001879666
Apoe	0.892219956	1.58E-02
Ugt1a7c	0.804616288	2.45E-02
Sbk1	0.801935131	3.46E-02
Slc15a2	0.790691302	4.66E-02
Nudt19	-0.763205418	0.005388158
Gbp9	-0.856412101	0.000339562
Kif20a	-1.041262607	3.60E-02
Slc6a17	-1.062205664	4.66E-02
Top2a	-1.075356208	0.016388164
Ugt1a5	-1.096587625	1.88E-03
Cenpe	-1.165400149	0.001879666
Slc22a30	-1.200051269	0.027843903
Mki67	-1.239031686	1.88E-03
Cenpf	-1.249956501	0.000520481
Knl1	-1.327898974	0.046551416
Serpinb8	-1.468102842	0.007847035
Kif20b	-1.543120204	0.0004933
Proser2	-2.068516959	1.11E-02
C130046K22Rik	-2.251553896	6.53E-03
Mansc4	-2.262278619	2.84E-02
Gbp10	-2.569837526	3.07636E-06
Glns-ps1	-3.078648463	2.23E-02
Gm4609	-4.91613781	0.022344019
Gbp11	-5.846008045	2.09E-02
Kdm5d	-9.658234454	4.66E-02

Supplemental Figure 8. TRAP-seq reveals differentially expressed genes (DEGs) in *Gak*-KO and C62 *Gak*-KO podocytes relative to WT control

(A-B) Top 30 upregulated (A) and downregulated (B) DEGs between *Gak*-KO and WT control podocytes from *in vivo* TRAP-seq. Analyzed via DESeq2. padj < 0.05. **(C)** Total upregulated and downregulated DEGs between C62 *Gak*-KO and WT podocytes from *in vivo* TRAP-seq. Analyzed via DESeq2. padj < 0.05.

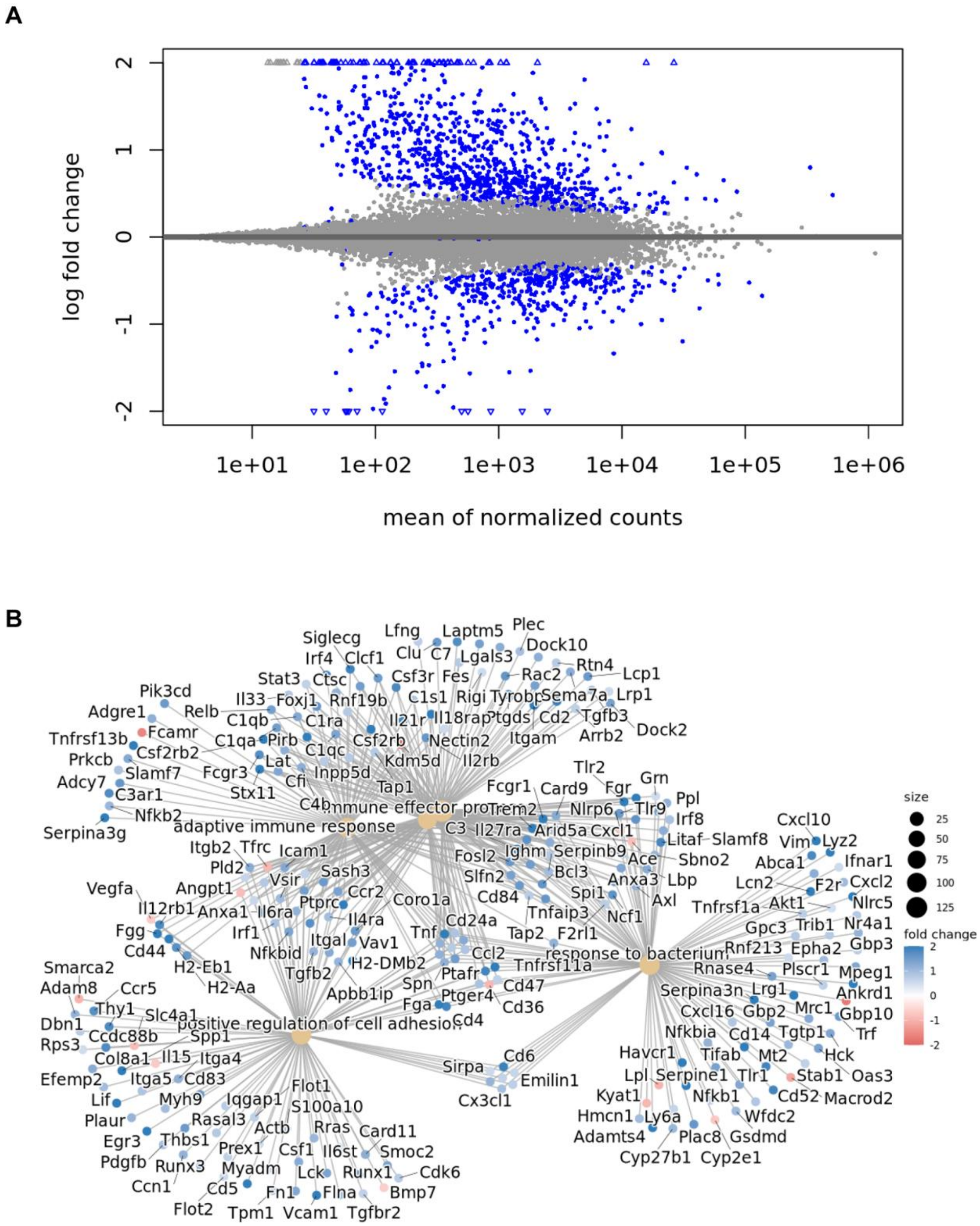

Supplemental Figure 9. Plots highlighting DEGs between *Gak*-KO and WT control podocytes

(A) MA scatter plot visualizing the log2 fold-change (M) relative to the average expression (A) of each gene across all samples. Blue color denotes padj < 0.1. Analyzed via DESeq2. **(B)** Cnet plot showing network of genes within the top 3 most enriched pathways between *Gak*-KO and WT podocytes. Analyzed using clusterProfiler.

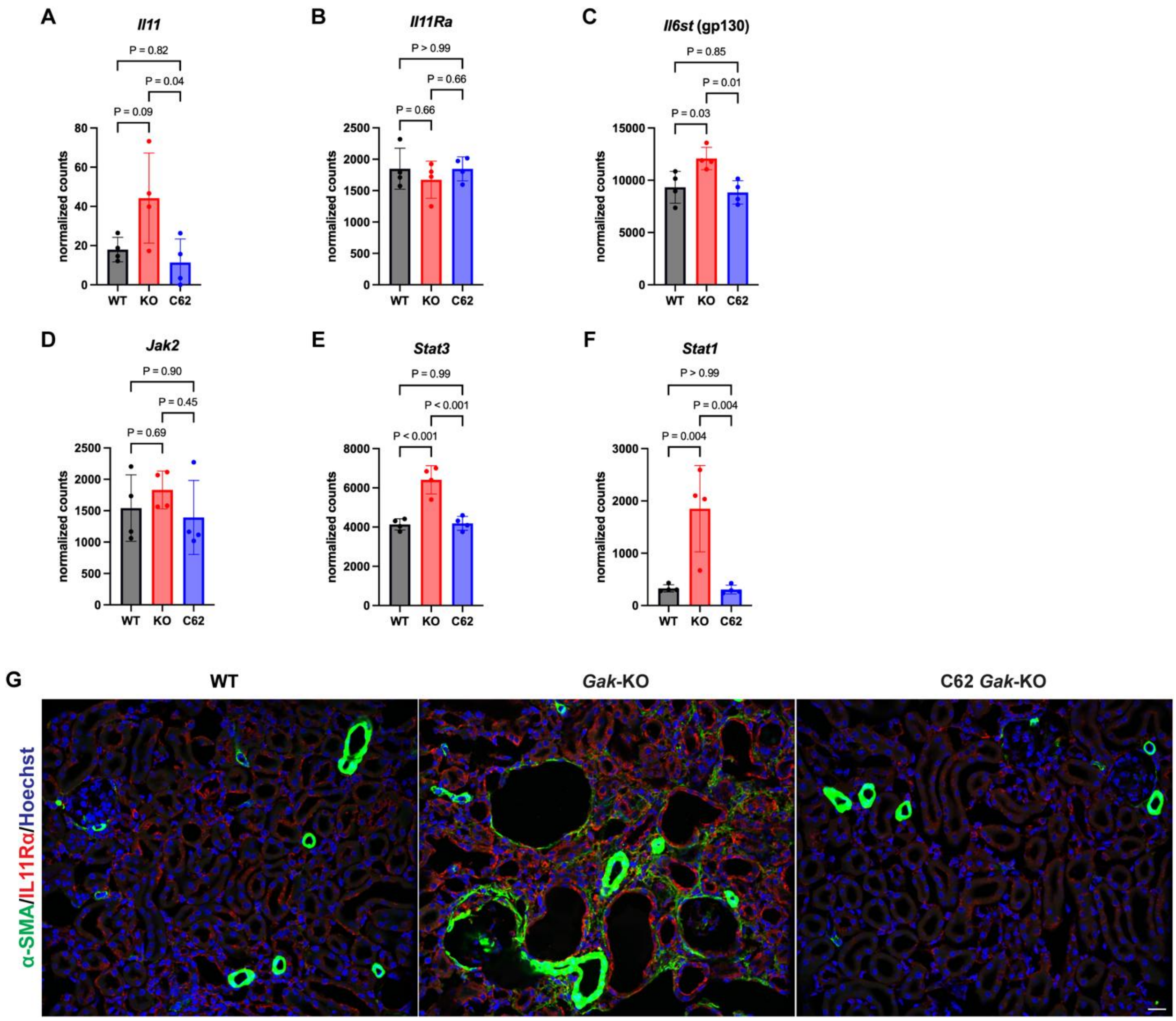

Supplemental Figure 10. Investigating potential autocrine and paracrine effects of IL-11 in primary podocytes

(A-F) Gene expression normalized counts of IL-11 signaling pathway members from TRAP-Seq experiment. Normalized using DESeq2. n = 4 biological replicates. One-way ANOVA with Tukey's multiple comparison test. **(G)** Representative immunofluorescence images of mouse kidney tissue sections co-stained with IL11Rα (red), α-SMA (green), and Hoechst (blue). Mice are 4 weeks of age. Scale bar = 20 μm.

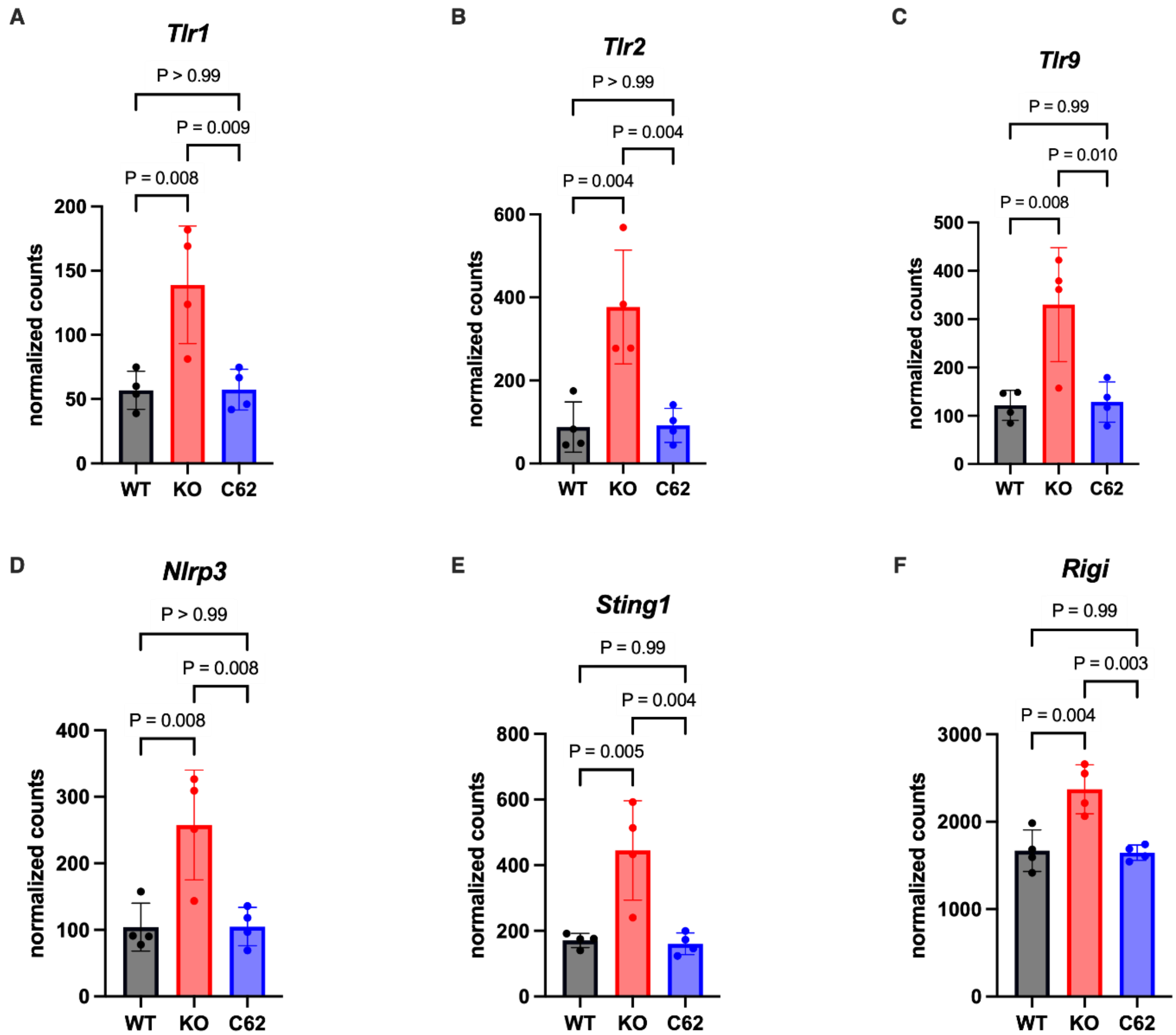

Supplemental Figure 11. Expression levels of pattern recognition receptor genes from TRAP-seq

(A-F) TRAP-seq experiment gene expression counts of various pattern recognition receptor genes. Normalized using DESeq2. n = 4 biological replicates. One-way ANOVA with Tukey's multiple comparison test.

Supplemental Table 1. List of primary antibodies and appropriate dilutions used across all experiments

Primary antibodies	Host	Catalog number	Dilution
anti-GAK	Rabbit	Gift from Dr. Lois Greene	WB 1:10000
anti-GAK	Rabbit	Sino Biological 203736-T42	IF 1:100
anti-GAPDH	Rabbit	Cell Signaling Technologies #2118	WB 1:2000
anti-LC3B	Mouse	Cell Signaling Technologies #83506	WB 1:1000; IF 1:200
anti-SYNPO (N-term)	Guinea pig	Progen GP94-N	IF 1:200
anti-WT1	Rabbit	Abcam ab89901	IF 1:200
anti-α-Tubulin	Mouse	Cell Signaling Technologies #3873	WB 1:1000
anti-RAB7	Rabbit	Cell Signaling Technologies #9367	IF 1:200
anti-AMPKα	Rabbit	Cell Signaling Technologies #2532	WB 1:1000
anti-phospho-AMPKα	Rabbit	Cell Signaling Technologies #4188	WB 1:1000
anti-mTOR	Rabbit	Abcam ab2732	WB 1:1000
anti-phospho-mTOR	Rabbit	Abcam ab109268	WB 1:1000
anti-ULK1	Rabbit	Sigma A7481	WB 1:1000
anti-phospho-ULK1 (Ser555)	Rabbit	Cell Signaling Technologies #5869	WB 1:1000
anti-phospho-ULK1 (Ser757)	Rabbit	Cell Signaling Technologies #14202	WB 1:1000
anti-LAMP1	Rabbit	Sigma-Aldrich L1418	WB 1:1000
anti-LAMP1	Mouse	Santa Cruz sc-20011	IF 1:100
anti-LAMP2	Rabbit	Cell Signaling Technologies #34141	WB 1:1000; IF 1:1500
anti-LAMP2	Rat	Developmental Studies Hybridoma Bank (ABL-93)	Immuno-EM 1:250
anti-Cathepsin B	Rabbit	Cell Signaling Technologies #31718	WB 1:1000
anti-Cathepsin D	Goat	Santa Cruz sc-6486	WB 1:250
anti-CI-MPR	Rabbit	Cell Signaling Technologies #14364	WB 1:1000
anti-Clathrin Light Chain	Rabbit	Milipore AB9884	IF 1:800
anti-RCAS1	Mouse	Santa Cruz sc-398052	IF 1:100
anti-α-SMA	Mouse	Invitrogen 53-9760-82	IF 1:150
anti-IL11Rα	Rabbit	Proteintech 10264-1-AP	IF 1:200

Supplemental Table 2. List of secondary antibodies and appropriate dilutions used across all experiments

Secondary antibodies	Host	Catalog number	Dilution
anti-mouse IgG, HRP-conjugated	Rabbit	Sigma-Aldrich AP160P	WB 1:3000
anti-rabbit IgG, HRP-conjugated	Goat	Sigma-Aldrich 12-348	WB 1:3000
anti-goat IgG, HRP-conjugated	Rabbit	Sigma-Aldrich AP106P	WB 1:3000
anti-goat IgG, Alexa Fluor 488	Donkey	Invitrogen A-11055	IF 1:200
anti-rabbit IgG, Alexa Fluor 488	Donkey	Invitrogen A-21206	IF 1:200
anti-rabbit IgG, Alexa Fluor 488	Goat	Invitrogen A-11008	IF 1:200
anti-rabbit IgG, Alexa Fluor 594	Donkey	Invitrogen A-21207	IF 1:200
anti-mouse IgG, Alexa Fluor 488	Donkey	Invitrogen A-21202	IF 1:200
anti-mouse IgG, Alexa Fluor 594	Donkey	Invitrogen A-21203	IF 1:200
anti-mouse IgG, Alexa Fluor 647	Donkey	Invitrogen A-31571	IF 1:200
anti-guinea pig IgG, Alexa Fluor 594	Goat	Invitrogen A-11073	IF 1:200
anti-rat IgG, Alexa Fluor 488-FluoroNanogold	Goat	Nanoprobes 7207-0.5ML	Immuno-EM 1:50

Supplemental Table 3. List of genotyping primer sequences

**Primer name**	**Sequence (5'-3')**
Gak FW	TGCCCTTTCAGCTCCCACACTCTTCTTCAC
Gak RV	GCAGCCAGCCTATGAGCCACAGACACTTAT
Gak C62 FW	CGCCTAAAGAAGAGGCTGTG
Gak C62 RV	CCACCTCAAAAACCAAGTCC
Pod-Cre FW	ACAGCTCCACCAAGACACAG
Pod-Cre RV	TCCGGTTATTCAACTTGCACC
Dtr FW	CACTGGATCTACGGACCAGC
Dtr RV	CGATTTTCCACTGGGAGGCT
TRAP(*L10*) FW	AGTGCTTCAGCCGCTACC
TRAP(*L10*) RV	CTTGCCAGCCTTGTTTAGG

Supplemental Methods

Antibodies and reagents

A detailed list of primary and secondary antibodies with catalog numbers and appropriate dilutions can be found in Supplemental Tables 1 and 2. Collagen type I (bovine) was purchased from Corning (354231) and used for coating cell culture dishes. Collagenase A was purchased from Roche (11088793001) and was used to digest kidney tissue for primary podocyte isolation. Diphtheria toxin (D0564) was obtained from Sigma-Aldrich and used for *in vitro* selection of primary podocytes. RPMI 1640 media (11875093) and Earle's balanced salt solution (EBSS; 24010043) were both purchased from Gibco and were used for regular and starvation media, respectively. Bafilomycin A1 was obtained from Cayman Chemicals (11038) and was used for autophagic flux experiments. LysoPrime Green and pHLys Red are part of the Lysosomal Acidic pH Detection Kit purchased from Dojindo (L266), which was utilized to examine lysosomal acidification. Hoechst 33342 was obtained from Biotium (40046) and was used as nuclear stain for immunofluorescence images.

Kidney histology and quantification

Mice were anesthetized by intraperitoneal injection of ketamine and xylazine, followed by circulatory perfusion through the left ventricle with ice-cold PBS, and followed by fixation with 4% paraformaldehyde (PFA). For histological analysis, fixed kidney samples were sent to the Yale Pathology Core Tissue Service for H&E, periodic acid–Schiff, and Masson's trichrome staining. To assess glomerulosclerosis, glomerular mesangial expansion, and the severity of renal tubulointerstitial lesions, kidney sections were assessed as previously described[1,2].

Transmission electron microscopy

For TEM, fixed kidney samples were sent to Yale Center for Cellular and Molecular Imaging (CCMI) Electron Microscopy Facility for sample preparation, sectioning, and imaging as previously described in our laboratory[2]. Briefly, solution containing 2.5% glutaraldehyde and 2% PFA in 0.1 M sodium cacodylate buffer at pH 7.4 was used to fix kidney tissue sections overnight at 4°C. Samples were post-fixed with 1% osmium tetroxide in 0.1 M cacodylate buffer for 1 hour and rinsed in distilled water prior to dehydration through an ethanol series. Next, dehydrated samples were transferred into a Leica 300 critical point dryer for approximately 3 hours. Samples were then carefully oriented and mounted on aluminum stubs. Lastly, images were taken using a FEI Tecnai T12 transmission electron microscope. The number of foot processes per area in each glomerular capillary were quantified using NIH ImageJ software as previously described[3].

Immuno-electron microscopy

Cultured cells are removed from the incubator, and culture medium is rapidly discarded, followed by fixation using a warm solution containing 4% paraformaldehyde (PFA) and 0.1% glutaraldehyde (GA) prepared in 0.1 M phosphate-buffered saline (PBS) at pH 7.4 for one hour at room temperature. The samples are then subjected to a PBS wash and treated with 20 mM glycine in PBS for 5 minutes. Blocking and permeabilization are performed using a solution of 4% bovine serum albumin (BSA) and 0.1% saponin in PBS for 30 minutes at room temperature, followed by two 5-minute rinses in 0.1% BSA prepared in PBS. Primary antibody incubation with LAMP2 (DSHB ABL-93; 1:250) is carried out in a solution containing 1% BSA and 0.1% saponin in PBS for 30 minutes at room temperature, followed by overnight incubation at 4 °C.

Samples are subsequently washed twice for 5 minutes each in PBS containing 0.1% BSA and 0.1% saponin. Secondary labeling is performed using Alexa Fluor 488-FluoroNanogold antibody (1:50) for 1 hour at room temperature, followed by two 5-minute rinses in 0.1% BSA in PBS and two additional 5-minute PBS washes. Post-fixation is accomplished using 2.5% glutaraldehyde in PBS for 1 hour at room temperature, after which the samples are rinsed twice with PBS and twice with distilled water for 5 minutes each. Silver enhancement is carried out using the Nanoprobes, Inc. enhancement solution for 15 minutes at 37 °C, and the reaction is terminated by three 5-minute rinses in distilled water. The samples are then exposed to 0.1 M sodium cacodylate buffer for 5 minutes, followed by treatment with 0.5% osmium tetroxide (OsO_4) prepared in 0.1 M sodium cacodylate for 30 minutes. Samples were then dehydrated with alcohol series 50%, 70%, 90%, and 100%, and embedded in Embed812 Epoxy resin (EMS. PA). 60nm ultrathin sections were collected with the Leica UC7 ultramicrotome and grids were post-stained with 2% uranyl acetate and led citrate before imaging with an 80kV FEI Technai Biotwin equipped with an AMT NanoSprint 15 Camera.

Biochemical measurement of urine albumin, urine creatinine, and plasma creatinine

Mice urine and plasma samples were collected weekly up to 6 weeks of age. Urine albumin was quantified using an albumin ELISA kit (Bethyl Laboratories Inc.) according to the manufacturer's instructions at an absorbance of 450 nm. Urine and plasma creatinine were measured using a colorimetric quantification kit (Bioassay Systems) at an absorbance of 490 nm. Results for both assays were obtained using a Synergy LX Multi-Mode Microplate Reader (BioTek).

Immunofluorescence

For immunocytochemistry, isolated primary podocytes were seeded on collagen I-coated 8-well chambered cover glass (Cellvis #C8-1.5P) designed for high resolution confocal imaging. Cells were washed 3 times with 1x PBS, followed by fixation with 4% PFA for 10 minutes. Cells were then washed with 1x PBS, and permeabilization with 0.1% Triton X-100 for 10 minutes. After another wash step with 1x PBS, samples were blocked with 5% BSA for 1 hour at room temperature. Cells were then incubated at 4°C overnight with an optimized dilution of appropriate primary antibodies at 4°C overnight. The next day, cells were washed 3 times with 1x PBS, followed by incubation with optimized dilutions of appropriate secondary antibodies. Both primary and secondary antibodies were diluted in 5% BSA in PBS. Cells were rinsed with 1x PBS 3 times and incubated in Hoechst (1:10,000) for 5 mins before imaging.

For tissue immunofluorescence, OCT-embedded kidneys were cryosectioned (5 µm) and mounted on charged glass slides. Samples were subjected to heat-mediated antigen retrieval using 10 mM sodium citrate buffer with 0.05% Tween 20 (pH 6.0) at 95°C for 10 minutes. Slides were allowed to cool down to room temperature before washing in 1x PBS 3 times. Samples were then blocked with 5% BSA for 1 hour at room temperature, followed by incubation with an optimized dilution of appropriate primary antibodies at 4°C overnight. The next day, slides were washed 3 times with 1x PBS, followed by incubation with optimized dilutions of appropriate secondary antibodies. Slides were rinsed with 1x PBS 3 times and incubated in Hoechst (1:10,000) for 5 mins before adding mounting media, applying coverslips, and sealing with clear nail polish.

All immunofluorescence images were acquired using an Andor CSU-WDi spinning disk confocal microscope equipped with a Nikon Ti-E CFI Plan Apochromat Lambda 40x, 60x, and 100x oil immersion objectives.

Western blotting

Primary podocytes were washed 3 times in ice-cold PBS and harvested with RIPA lysis buffer using a plastic cell scraper. Protease inhibitor cocktail (Roche 11836170001) and phosphatase inhibitor cocktail (Sigma P5726-5ML) were both added to the RIPA buffer to inhibit protein degradation. Protein concentrations were quantified using the Pierce BCA Protein Assay Kit (Thermo Fisher 23225). Samples were diluted to appropriate concentrations in 4x Laemmli buffer (Bio-Rad 1610747) and denatured for 10 mins on a 95°C heat block. Equal amounts of protein were loaded and ran on 10% or 15% acrylamide gels, depending on the molecular weight of target protein. Samples were then transferred onto a PVDF membrane (Milipore IPVH00010), followed by blocking with 5% nonfat milk or 5% BSA in TBST. Membranes were then incubated with optimized dilutions of appropriate primary antibodies overnight at 4°C on a shaker. After several washes in TBST, membranes were incubated with appropriate HRP-conjugated secondary antibodies. Signals were detected using Clarity Western ECL Substrate (Bio-Rad 1705061) and imaged using Odyssey Fc (LI-COR Biosciences). For quantification, densitometry analysis was performed using NIH ImageJ software.

Cell adhesion assay

Adhesion assay was performed as previously described[4]. Briefly, primary podocytes were harvested using 0.05% Trypsin-EDTA (Gibco 25300054). Equal number of cells were plated and

allowed to adhere on collagen I-coated 96-well plates for 2 hours (5,000 cells per well). Cells were washed 3 times with 1x PBS to remove unattached cells, followed by fixation with 95% ethanol for 15 minutes. After washing in PBS, cells were stained with 0.1% crystal violet for 15 minutes at room temperature with gentle shaking. The plate was washed in distilled water 5 times to remove excess crystal violet. Cells were then lysed in 0.2% Triton X-100 while shaking, until a uniform color is obtained. Results were measured at an absorbance of 595 nm using a Synergy LX Multi-Mode Microplate Reader (BioTek).

Labeled transferrin uptake assay

Cultured primary podocytes were washed 2 times with 1x PBS followed by incubation in serum-free media for 1 hour. Cells were then incubated with Alexa Fluor 488-conjugated Mouse Transferrin (Jackson Immunoresearch 015-600-050) at a working concentration of 30 µg/mL diluted in serum-free media. Cells were washed 3 times with 1x PBS followed by incubation in Hoechst (1:10,000) for 5 mins before imaging.

TRAP-seq

Podocyte mRNA was isolated from 3- to 4-week-old mice as previously described[2,5,6]. To obtain enough podocyte mRNA for RNA-Seq analysis, each sample was obtained from pooled tissue samples from 4-5 mice. Briefly, mice were anesthetized and perfused with RNase-free 1X PBS through the left ventricle. Kidneys were harvested, and the cortex was carefully dissected, minced into small pieces, and transferred to a prechilled tissue homogenizing tube (Precellys CK28-R) containing lysis buffer, followed by gentle homogenization (Precellys Evolution, Bertin Technologies). NP-40 and 1,2-diheptanoyl-sn-glycero-3-phosphocholine (850306P-1g,

Avanti Polar Lipids Inc.) were added, followed by centrifugation at 2,000g and 17,200g at 4°C for 10 minutes, respectively. GFP-tagged ribosomes from tissue lysate were isolated by overnight immunoprecipitation at 4°C using Streptavidin Myone T1 dynabeads (Invitrogen 65601) coupled with high-affinity GFP antibodies: Htz-GFP-19C8 and Htz-GFP-19F7 (Antibody and Bioresource Core Facility, Memorial Sloan-Kettering Cancer Center). After a series of wash steps, the bound RNA fraction was extracted from the magnetic beads using RLT lysis buffer containing β-mercaptoethanol. The resulting supernatant was cleaned using the RNeasy MiniElute Cleanup Kit (Qiagen) following the manufacturer's instructions. Resulting mRNA samples were sent to the Yale Center for Genomic Analysis (YCGA) for ribosomal reduction to efficiently remove the rRNA component within each sample, followed by RNA-seq.

Raw fastq files were converted to raw counts using a standard pipeline provided by YCGA. This pipeline uses the following tools: fastp[7] for quality control and filtering, HISAT2[8] for splice-aware alignment of reads to the genome, and Stringtie[8] for quantification. The resulting counts table was analyzed using DESeq2[9] to obtain DEGs between samples. Functional analysis was performed using clusterProfiler 4.0[10] to identify enriched pathways from the list of DEGs.

Cytokine profiling

Cells were plated in 10 cm^2 dishes and incubated in serum-free media for 16 hours. Cell culture supernatants (~8 mL) were then collected and filtered through 0.45 μm PVDF syringe filter to remove cellular debris. Cell culture supernatants were spun down and concentrated using Amicon Ultra Centrifugal Filter, 3 kDa (UFC900308) at 4000 g for 1 hour at room temperature. Phosphatase and protease inhibitors were added to the resulting concentrated samples (~300 uL)

before being sent to Eve Technologies for cytokine profiling. In this study, we used Luminex® xMAP® technology to quantitatively and simultaneously detect forty-four mouse cytokines, chemokines and growth factors.

The multiplexing analysis was performed by Eve Technologies Corporation (Calgary, Alberta, Canada) using the Luminex® 200™ system (Luminex Corporation/DiaSorin, Saluggia, Italy) with Bio-Plex Manager™ software (Bio-Rad Laboratories Inc., Hercules, California, United USA). Forty-four markers were measured in the samples using two separate Eve Technologies' panels. The 44-plex consisted of Eotaxin/CCL11, Erythropoeitin (EPO), Exodus2/6Ckine, Fractalkine/CX3CL1, G-CSF/CSF-3, GM-CSF, GROα/CXCL1/KC/CINC-1, GROβ/CXCL2/MIP-2/CINC-3, IFN-γ, IFN-β, IL-1α, IL-1β, IL-2, IL-3, IL-4, IL-5, IL-6, IL-7, IL-9, IL-10, IL-11, IL-12(p40), IL-12(p70), IL-13, IL-15, IL-16, IL-17, IL-20, IP-10/CXCL10, LIF, LIX, MCP-1/CCL2, MCP-5/CCL12, M-CSF, MDC/CCL22, MIG/CXCL9, MIP-1α/CCL3, MIP-1β/CCL4, MIP-3α/CCL20, MIP-3β/CCL19, RANTES/CCL5, TARC/CCL17, TNF-α and VEGF-A. Assay sensitivities of these markers range from 0.3 – 30.6 pg/mL. Individual analyte sensitivity values are available in the MilliporeSigma MILLIPLEX® protocols.

Study approval

All animal experiments were carried out in accordance with the principles and procedures outlined in the NIH's Guide for the Care and Use of Laboratory Animals and approved by the Institutional Animal Care and Use Committee at Yale University.

References

1. Lausecker F, Tian X, Inoue K, et al. Vinculin is required to maintain glomerular barrier integrity. *Kidney International*. 2018;93(3):643-655. doi:10.1016/j.kint.2017.09.021
2. Tian X, Pedigo CE, Li K, et al. Profilin1 is required for prevention of mitotic catastrophe in murine and human glomerular diseases. *J Clin Invest*. 2023;133(24). doi:10.1172/JCI171237
3. Tian X, Inoue K, Zhang Y, et al. Inhibiting calpain 1 and 2 in cyclin G associated kinase–knockout mice mitigates podocyte injury. *JCI Insight*. 2020;5(22). doi:10.1172/jci.insight.142740
4. Ishibe S, Joly D, Liu ZX, Cantley LG. Paxillin Serves as an ERK-Regulated Scaffold for Coordinating FAK and Rac Activation in Epithelial Morphogenesis. *Molecular Cell*. 2004;16(2):257-267. doi:10.1016/j.molcel.2004.10.006
5. Wang Y, Niu A, Pan Y, et al. Profile of Podocyte Translatome During Development of Type 2 and Type 1 Diabetic Nephropathy Using Podocyte-Specific TRAP mRNA RNA-seq. *Diabetes*. 2021;70(10):2377-2390. doi:10.2337/db21-0110
6. Heiman M, Kulicke R, Fenster RJ, Greengard P, Heintz N. Cell type–specific mRNA purification by translating ribosome affinity purification (TRAP). *Nat Protoc*. 2014;9(6):1282-1291. doi:10.1038/nprot.2014.085
7. Chen S, Zhou Y, Chen Y, Gu J. fastp: an ultra-fast all-in-one FASTQ preprocessor. *Bioinformatics*. 2018;34(17):i884-i890. doi:10.1093/bioinformatics/bty560
8. Pertea M, Kim D, Pertea GM, Leek JT, Salzberg SL. Transcript-level expression analysis of RNA-seq experiments with HISAT, StringTie and Ballgown. *Nat Protoc*. 2016;11(9):1650-1667. doi:10.1038/nprot.2016.095
9. Love MI, Huber W, Anders S. Moderated estimation of fold change and dispersion for RNA-seq data with DESeq2. *Genome Biology*. 2014;15(12):550. doi:10.1186/s13059-014-0550-8
10. Wu T, Hu E, Xu S, et al. clusterProfiler 4.0: A universal enrichment tool for interpreting omics data. *The Innovation*. 2021;2(3):100141. doi:10.1016/j.xinn.2021.100141
